# Author Correction: A previously uncharacterized Factor Associated with Metabolism and Energy (FAME/C14orf105/CCDC198/1700011H14Rik) is related to evolutionary adaptation, energy balance, and kidney physiology

**DOI:** 10.1038/s41467-023-39373-w

**Published:** 2023-06-15

**Authors:** Julian Petersen, Lukas Englmaier, Artem V. Artemov, Irina Poverennaya, Ruba Mahmoud, Thibault Bouderlique, Marketa Tesarova, Ruslan Deviatiiarov, Anett Szilvásy-Szabó, Evgeny E. Akkuratov, David Pajuelo Reguera, Hugo Zeberg, Marketa Kaucka, Maria Eleni Kastriti, Jan Krivanek, Tomasz Radaszkiewicz, Kristína Gömöryová, Sarah Knauth, David Potesil, Zbynek Zdrahal, Ranjani Sri Ganji, Anna Grabowski, Miriam E. Buhl, Tomas Zikmund, Michaela Kavkova, Håkan Axelson, David Lindgren, Rafael Kramann, Christoph Kuppe, Ferenc Erdélyi, Zoltán Máté, Gábor Szabó, Till Koehne, Tibor Harkany, Kaj Fried, Jozef Kaiser, Peter Boor, Csaba Fekete, Jan Rozman, Petr Kasparek, Jan Prochazka, Radislav Sedlacek, Vitezslav Bryja, Oleg Gusev, Igor Adameyko

**Affiliations:** 1 Department of Orthodontics, University Leipzig Medical Center, Leipzig, Germany; 2 CeMM Research Center for Molecular Medicine of the Austrian Academy of Sciences, 1090 Vienna, Austria; 3 Ludwig Boltzmann Institute for Rare and Undiagnosed Diseases, 1090 Vienna, Austria; 4 Department of Neuroimmunology, Center for Brain Research, Medical University Vienna, Vienna, Austria; 5 Central European Institute of Technology, Brno University of Technology, Brno, Czech Republic; 6 Regulatory Genomics Research Center, Institute of Fundamental Medicine and Biology, Kazan Federal University, Kazan, Russia; 7 Endocrinology Research Center, Moscow, Russia; 8 Laboratory of Integrative Neuroendocrinology, Institute of Experimental Medicine, 1083 Budapest, Hungary; 9 Department of Applied Physics, Royal Institute of Technology, Science for Life Laboratory, 171 65, Stockholm, Sweden; 10 University of Oxford, MRC Weatherall Institute of Molecular Medicine, Radcliffe Department of Medicine, Oxford, OX3 9DS UK; 11 Institute of Molecular Genetics of the Czech Academy of Science, Czech Centre for Phenogenomics, Vestec, Czech Republic; 12 Department of Neuroscience, Karolinska Institutet, Stockholm, Sweden; 13 Department of Physiology and Pharmacology, Karolinska Institutet, Stockholm, Sweden; 14 Max Planck Institute for Evolutionary Biology, Plön, 24306 Germany; 15 Department of Histology and Embryology, Faculty of Medicine, Masaryk University, Brno, Czech Republic; 16 Institute of Experimental Biology, Faculty of Science, Masaryk University, Brno, Czech Republic; 17 Central European Institute of Technology, Masaryk University, Brno, Czech Republic; 18 Institute of Pathology & Electron Microscopy Facility, RWTH Aachen University Hospital, Aachen, Germany; 19 Translational Cancer Research, Department of Laboratory Medicine, Lund University, Medicon Village, Scheelevägen 2, Lund, Sweden; 20 Institute of Experimental Medicine and Systems Biology, RWTH Aachen University, Aachen, Germany; 21 Medical Gene Technology Unit, Institute of Experimental Medicine, Budapest, Hungary; 22 Department of Molecular Neurosciences, Center for Brain Research, Medical University Vienna, Vienna, Austria; 23 Luxembourg Centre for Systems Biomedicine, University of Luxembourg, 6, avenue du Swing, 4367 Belvaux, Luxembourg; 24 Intractable Disease Research Center, Graduate School of Medicine, Juntendo University, Tokyo, Japan

**Keywords:** Evolutionary genetics, Kidney, Genetic models

Correction to: *Nature Communications* 10.1038/s41467-023-38663-7 published online 29 May 2023

The original version of this Article incorrectly listed Prof. Oleg Gusev as being affiliated with “Endocrinology Research Center, Moscow, Russia” and “Center for Integrative Medical Sciences, RIKEN, Yokohama City, Kanagawa, Japan”. The aforementioned affiliations have now been removed and the following was added for Prof. Oleg Gusev “Regulatory Genomics Research Center, Institute of Fundamental Medicine and Biology, Kazan Federal University, Kazan, Russia” . In addition, information was missing from the Funding section and has now been added to the acknowledgements section which reads “OG and RD were supported by Ministry of Science and Higher Education of the Russian Federation grant 075-15-2021-1344 and Japan Society for the Promotion of Science (JSPS) KAKENHI JP23H02226.”.

These errors have been corrected in both the PDF and HTML versions of the Article.

