# Supplementary material for: A previously uncharacterized Factor Associated with Metabolism and Energy (FAME/C14orf105/CCDC198/1700011H14Rik) is related to evolutionary adaptation, energy balance, and kidney physiology
Source: Nat Commun. 2023 May 29;14:3092. doi: 10.1038/s41467-023-38663-7 (PMC10226981; doi:10.1038/s41467-023-38663-7)
Supplement: Supplementary file 1 — Supplementary Information [file 41467_2023_38663_MOESM1_ESM.pdf]

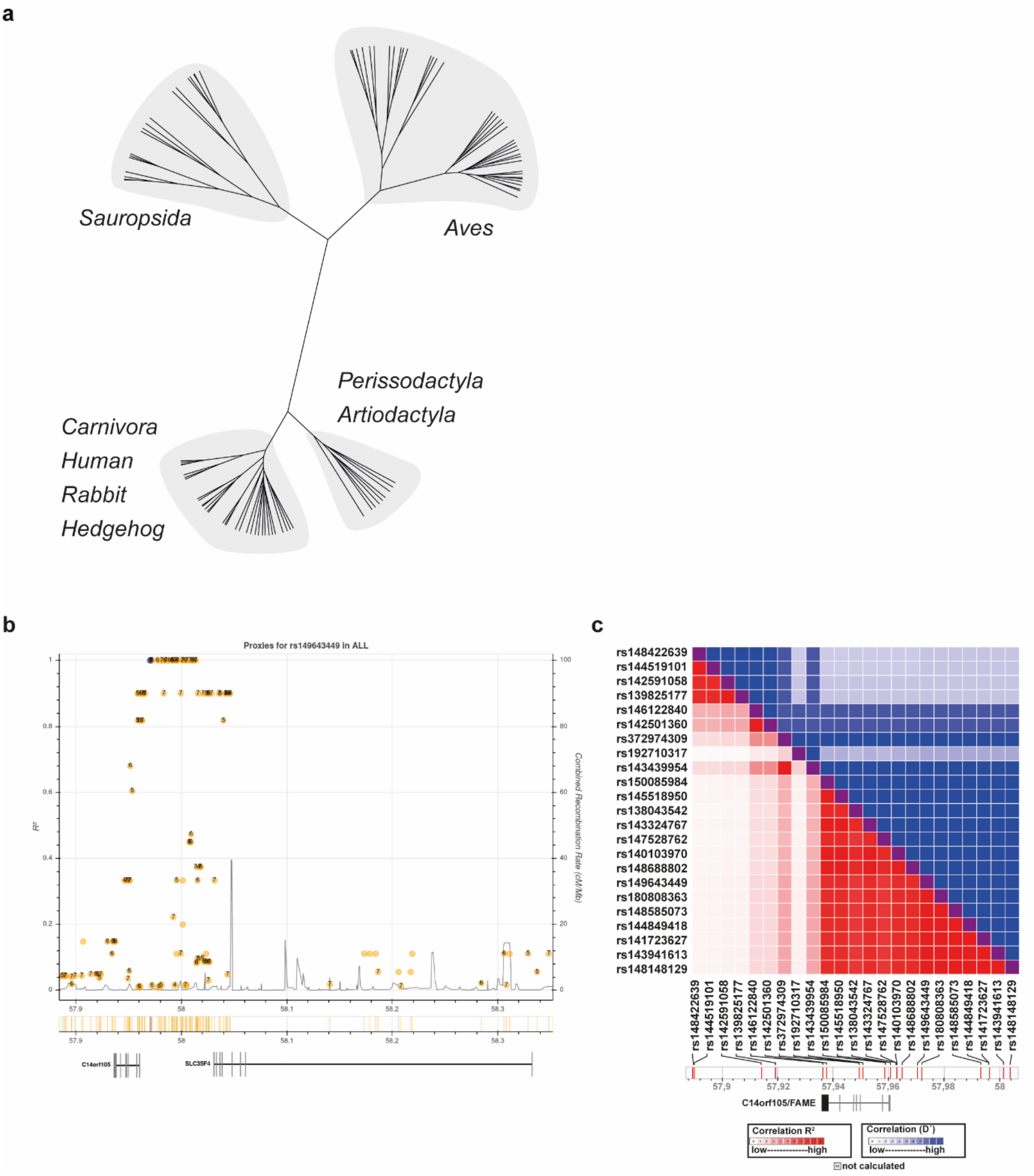

Supplementary Figure 1: Comparative genomics analysis of *FAME/C14orf105*

(a) Phylogenetic tree for FAME orthologs of diverse species. (**b**) Linkage disequilibrium between the polymorphisms in **Figure 1f**. Fourteen of these alleles form a block with linkage disequilibrium $r^2 > 0.8$. (**c**) Introgressed Neanderthal haplotype encompassing *C14orf105.* 58 SNPs form a haplotype ($r^2 > 0.8$), which is tagged by rs149643449, spanning the genomic region chr14:57958614-58046101. **(b-c)** Figure generated using LDlink (Machiela and Chanock 2015) using data from the 1000 Genomes Project (Genomes Project, Auton et al. 2015). The abscissa gives genomic coordinates on chromosome 14 (*hg19*).

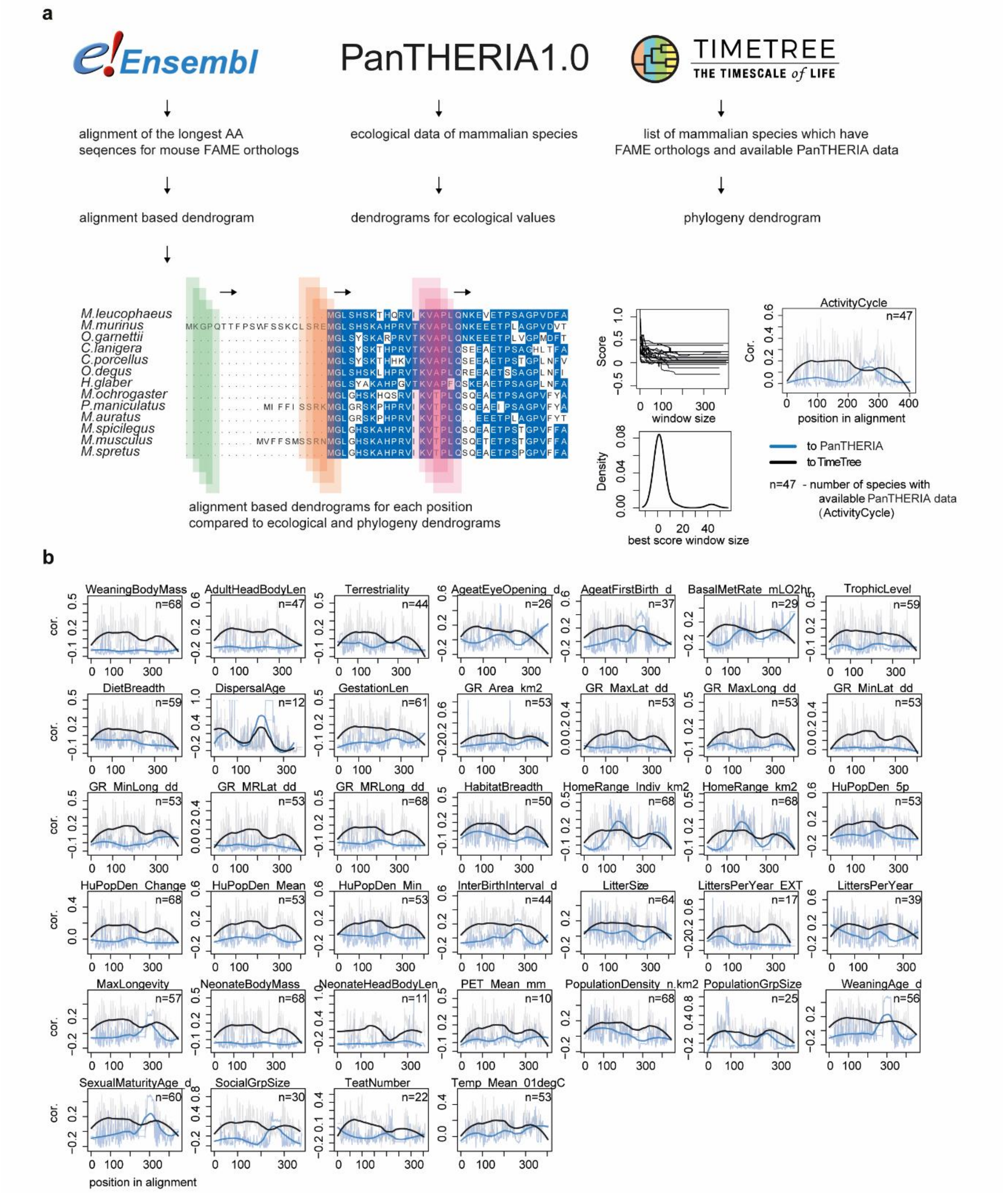

Supplementary Figure 2: FAME evolution with animal lifestyle

(**a**) Basic scheme of the correlation pipeline. Orthologous gene IDs for *Mus musculus Fame* were obtained from Ensembl. For each gene and species, the longest amino acid (AA) sequences were downloaded through biomaRt. Ecological data for mammalian species were obtained from PanTHERIA1.0. Phylogenetic distances of species used as controls were obtained from TimeTree database. For each condition in PanTHERIA (n=44) a subset of species with available data was extracted and an ecologic dendrogram was built based on the feature scores. A subset of the phylogenetic tree was created for selected species. Amino acid alignments were created for the subset list. The window size was analyzed to provide the

highest difference between cophenetic correlation of the alignment k-mer dendrogram with ecologic and phylogenetic dendrograms. As expected, this score drops with increasing k-mer length and, in most cases, the shortest 2-mer window provides the highest difference (density plot). Scanning the alignment subset (n=47) with the 2 AA window for the feature "ActivityCycle" resulted in trends overlapping with phylogeny. (**b**) Further examples of correlation trends are shown. Detailed explanation of ecologic factors titles can be accessed under [https://esapubs.org/archive/ecol/E090/184/metadata.htm]. Interestingly, the sites of ties can be the same or different and trends in general have different trajectories leading to functional significance or insignificance of protein domains in relation to ecological factor.

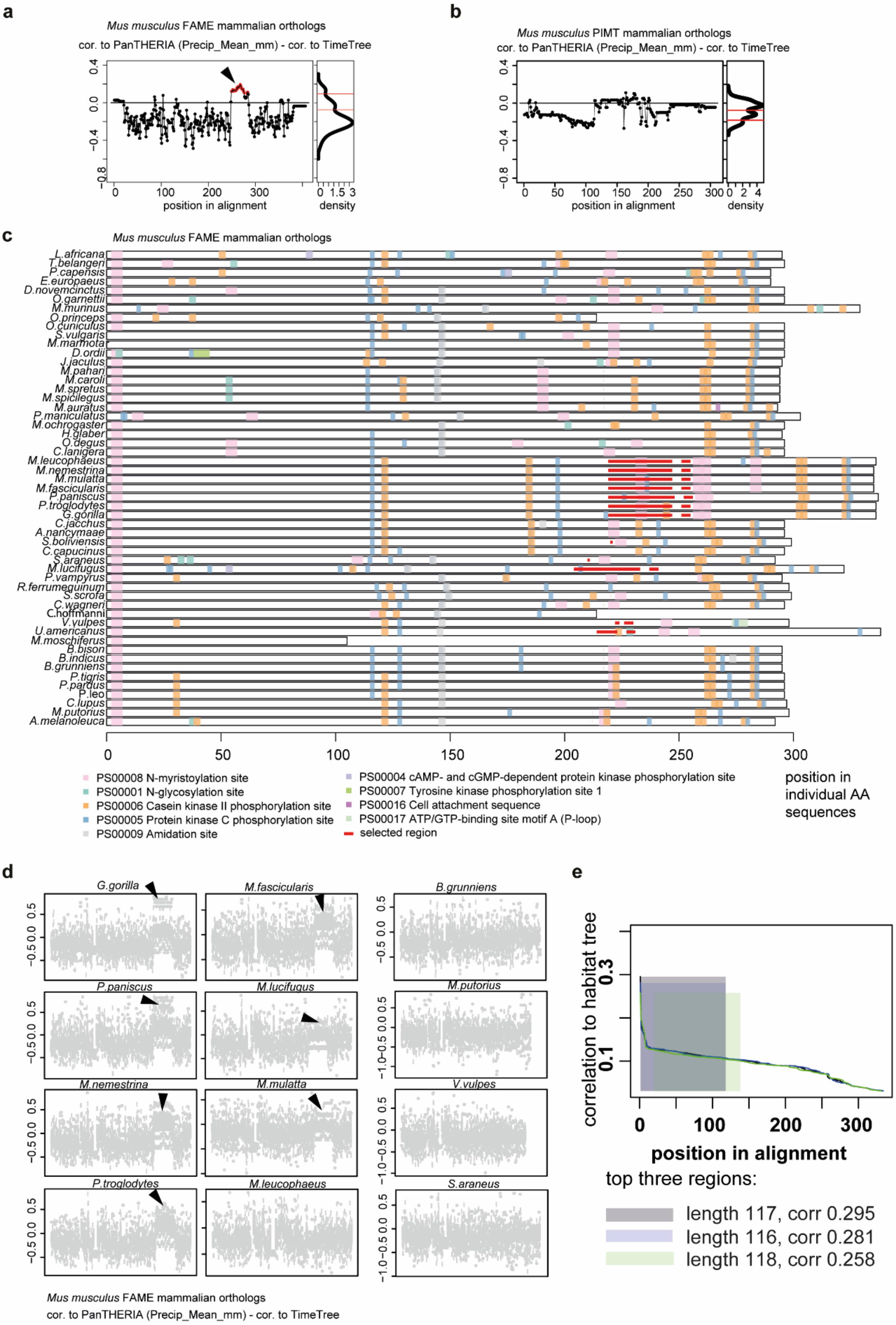
a
Mus musculus FAME mammalian orthologs
cor. to PanTHERIA (Precip_Mean_mm) - cor. to TimeTree
position in alignment
density
b
Mus musculus PIMT mammalian orthologs
cor. to PanTHERIA (Precip_Mean_mm) - cor. to TimeTree
position in alignment
density
c
Mus musculus FAME mammalian orthologs
position in individual AA sequences
PS00008 N-myristoylation site
PS00001 N-glycosylation site
PS00006 Casein kinase II phosphorylation site
PS00005 Protein kinase C phosphorylation site
PS00009 Amidation site
PS00004 cAMP- and cGMP-dependent protein kinase phosphorylation site
PS00007 Tyrosine kinase phosphorylation site 1
PS00016 Cell attachment sequence
PS00017 ATP/GTP-binding site motif A (P-loop)
selected region
d
G.gorilla
M.fascicularis
B.grunniens
P.paniscus
M.lucifugus
M.putorius
M.nemestrina
M.mulatta
V.vulpes
P.troglodytes
M.leucophaeus
S.araneus
Mus musculus FAME mammalian orthologs
cor. to PanTHERIA (Precip_Mean_mm) - cor. to TimeTree
e
correlation to habitat tree
position in alignment
top three regions:
length 117, corr 0.295
length 116, corr 0.281
length 118, corr 0.258

Supplementary Figure 3: Comparison of FAME orthologs

(**a**) FAME orthologs contain high scoring regions (2-mer alignment based dendrogram cor. to PanTHERIA - cor. to TimeTree) between 200 and 300 AA of the alignment. We extracted these positions by selecting minimums for density of positions and using the highest minimum as a threshold (0.095, top red line). (**b**) Mammalian PIMT orthologs (n=52) were selected as negative controls and checked for difference between correlation profiles. In general, score values are lower and most of them are close to zero (highest threshold matches with negative score value -0.075, top red line). This can be explained as an absence of functional connections of any PIMT domains with precipitation. (**c**) Transferring of selected region from the alignment to individual molecules. Prosite domains are annotated and labeled as boxes with different colors. Red color indicates the highest scoring region. Approximately 10 proteins contain this annotated region, and it overlaps with Prosite domains. Overlap significance (Fisher's exact test) highlights the match with the N-myristylation site (PS00008) for the majority of proteins (Supplementary Data 6). (**d**) Repeating the experiment with 100 bootstraps for 10 random sequences. Arrows points out the high scoring region of individual molecules. (**e**) Correlation between alignments and habitat tree for the top three windows.

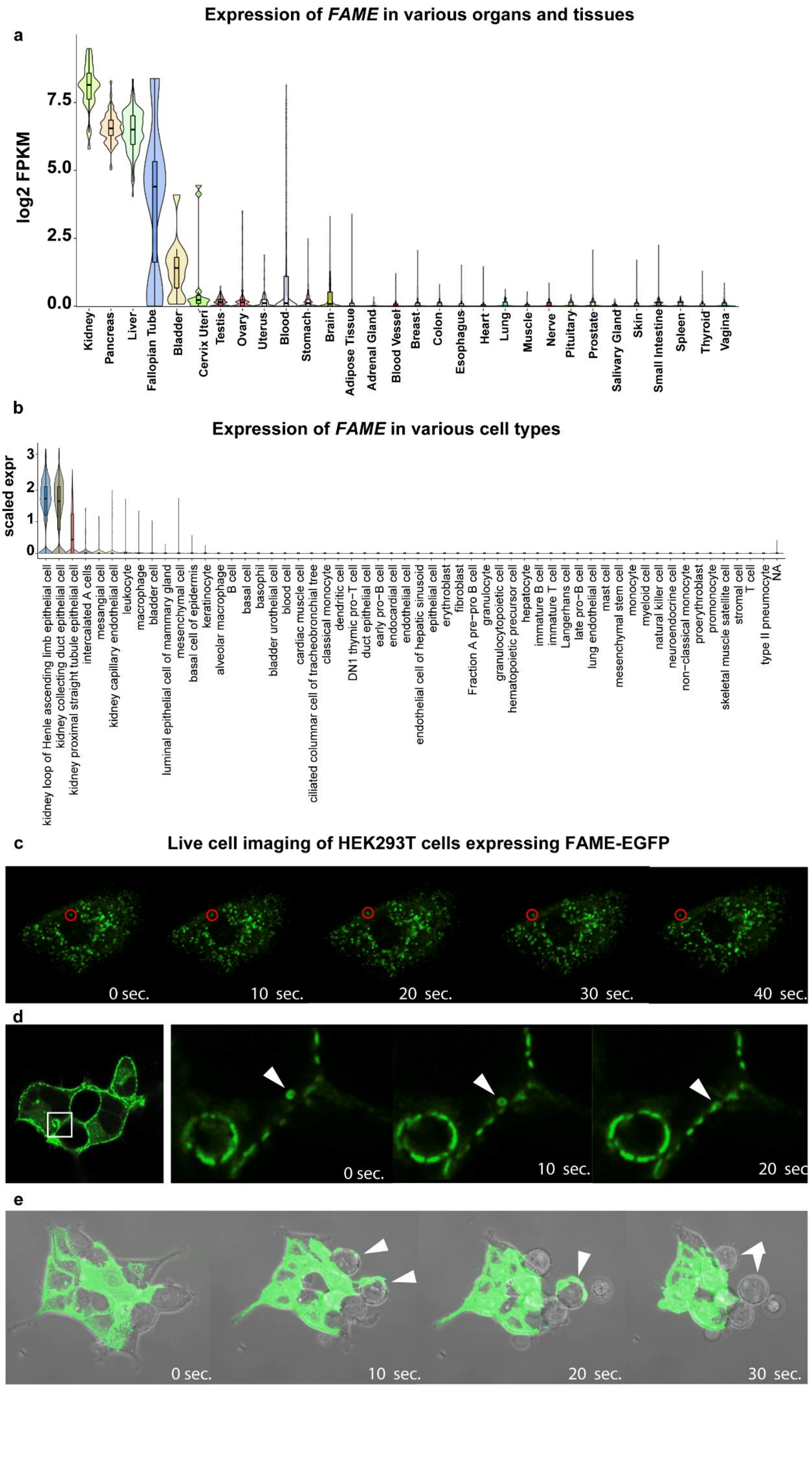
a
Expression of FAME in various organs and tissues
log2 FPKM
7.5
5.0
2.5
0.0
Kidney
Pancreas
Liver
Fallopian Tube
Bladder
Cervix Uteri
Testis
Ovary
Uterus
Blood
Stomach
Brain
Adipose Tissue
Adrenal Gland
Blood Vessel
Breast
Colon
Esophagus
Heart
Lung
Muscle
Nerve
Pituitary
Prostate
Salivary Gland
Skin
Small Intestine
Spleen
Thyroid
Vagina
b
Expression of FAME in various cell types
scaled expr
3
2
1
0
kidney loop of Henle ascending limb epithelial cell
kidney collecting duct epithelial cell
kidney proximal straight tubule epithelial cell
intercalated A cells
mesangial cell
kidney capillary endothelial cell
leukocyte
macrophage
bladder cell
luminal epithelial cell of mammary gland
mesenchymal cell
basal cell of epidermis
keratinocyte
alveolar macrophage
B cell
basal cell
basophil
bladder urothelial cell
blood cell
cardiac muscle cell
ciliated columnar cell of tracheobronchial tree
classical monocyte
dendritic cell
DN1 thymic pro−T cell
duct epithelial cell
early pro−B cell
endocardial cell
endothelial cell
endothelial cell of hepatic sinusoid
epithelial cell
erythroblast
fibroblast
Fraction A pre−pro B cell
granulocyte
granulocytopoietic cell
hematopoietic precursor cell
hepatocyte
immature B cell
immature T cell
Langerhans cell
late pro−B cell
lung endothelial cell
mast cell
mesenchymal stem cell
monocyte
myeloid cell
natural killer cell
neuroendocrine cell
non−classical monocyte
proerythroblast
promonocyte
skeletal muscle satellite cell
stromal cell
T cell
type II pneumocyte
NA
c
Live cell imaging of HEK293T cells expressing FAME-EGFP
0 sec.
10 sec.
20 sec.
30 sec.
40 sec.
d
0 sec.
10 sec.
20 sec
e
0 sec.
10 sec.
20 sec.
30 sec.

Supplementary Figure 4: *FAME* expression in tissues and protein localization upon overexpression

(**a**) Analysis of *FAME* expression in various human tissues based on Genotype-Tissue Expression data. (**b**) Detailed expression analysis of *Fame* in different mouse cell types using Tabula muris, a recently derived mouse single cell atlas. (**a-b**) Descriptive statistics can be accessed in Supplementary Data 13. **(c-e)** Live cell imaging of overexpressed FAME tagged with EGFP in HEK293T cells (**c**) FAME protein localizes to fast trafficking vesicular structures. Red circle follows a single vesicle over time. (**d**) FAME positive vesicle are transported amongst cells (white arrow). **(e)** $FAME^{+}$ cells share membranes with FAME cells (white arrows).

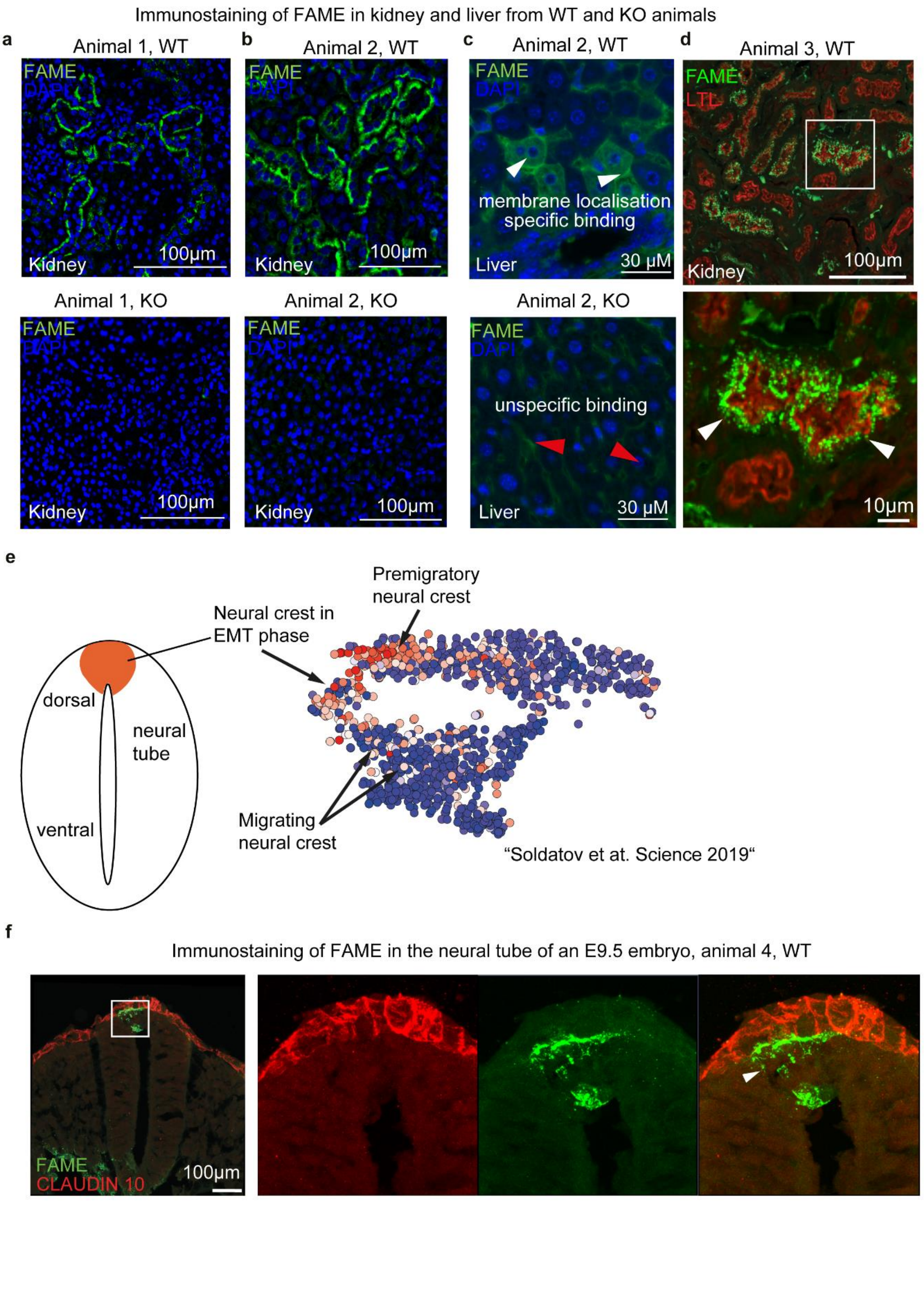

Immunostaining of FAME in kidney and liver from WT and KO animals
a
Animal 1, WT
FAME
DAPI
Kidney
100μm
Animal 1, KO
FAME
DAPI
Kidney
100μm
b
Animal 2, WT
FAME
DAPI
Kidney
100μm
Animal 2, KO
FAME
DAPI
Kidney
100μm
c
Animal 2, WT
FAME
DAPI
membrane localisation
specific binding
Liver
30 μM
Animal 2, KO
FAME
DAPI
unspecific binding
Liver
30 μM
d
Animal 3, WT
FAME
LTL
Kidney
100μm
10μm
e
Premigratory
neural crest
Neural crest in
EMT phase
dorsal
neural
tube
ventral
Migrating
neural crest
"Soldatov et at. Science 2019"
f
Immunostaining of FAME in the neural tube of an E9.5 embryo, animal 4, WT
FAME
CLAUDIN 10
100μm

Supplementary Figure 5: Endogenous localization and appearance of FAME in the adult mouse and developing embryo

(a-b) Knockout validation of the used FAME mouse monoclonal antibody. Protein specificity of the antibody is shown by immunohistochemistry on paraffin embedded tissue sections. Note the absence of signal in the two independent knockout (KO) animals. DAPI (blue), FAME (green) **(c)** Unspecific signal, often in the form of pronounced puncta, was frequently observed both in wild type in KO animals. **(a-c)** Representative images from 3 individual animals for each genotype. **(d)** Cell type specific staining was confirmed in high-magnification images, as shown here in the mouse kidney counterstained with lotus tetragonolobus lectin (LTL, red) visualizing proximal tubules. Note the vesicular appearance of FAME that corresponds to overexpression data. **(e)** Single cell analysis of murine neural crest and experimental validations associate *Fame* with epithelial-to-mesenchymal transition (EMT) (Soldatov, Kaucka et al. 2019). Schematic representation of the neural tube during development. **(f)** Transversal cryosection of an E9.5 mouse embryo with an immunofluorescence staining of the neural tube for FAME and CLAUDIN10. The white arrow points to an area of high FAME expression within the dorsal neural tube. Representative image from 3 tissue sections.

HEK293T cells transfected with FAME-pEGFP-N1 and stained with anti-FAME Antibody (without heat antigen retrieval)

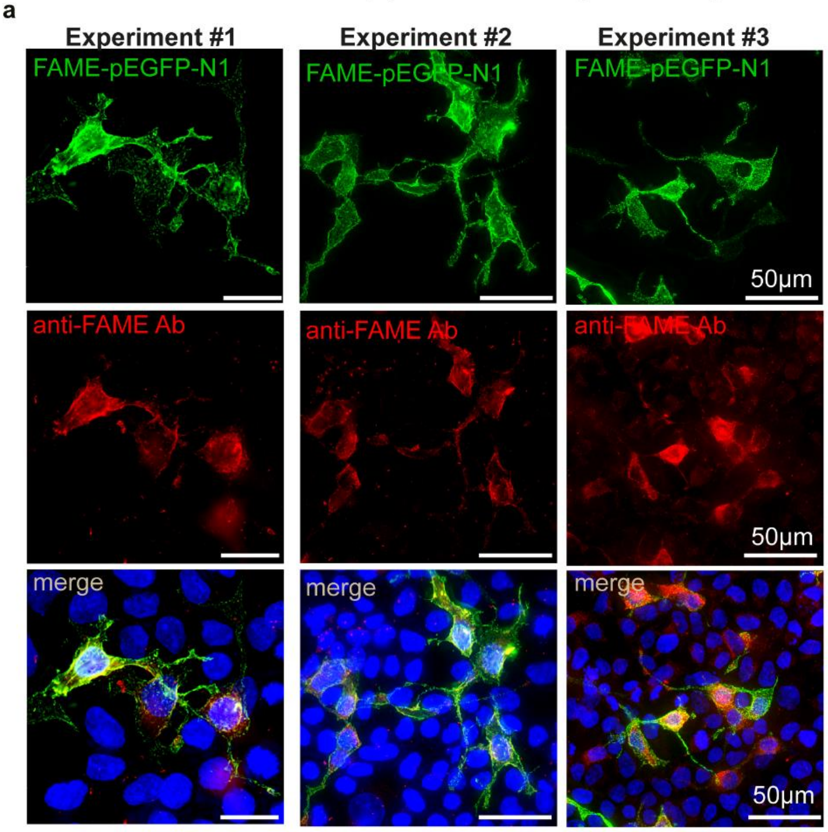

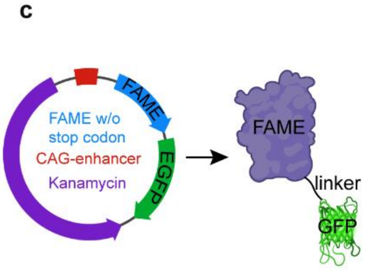

HEK293T cells transfected with FAME-pEGFP-N1 and stained with anti-GFP and anti-FAME Antibody after heat antigen retrieval

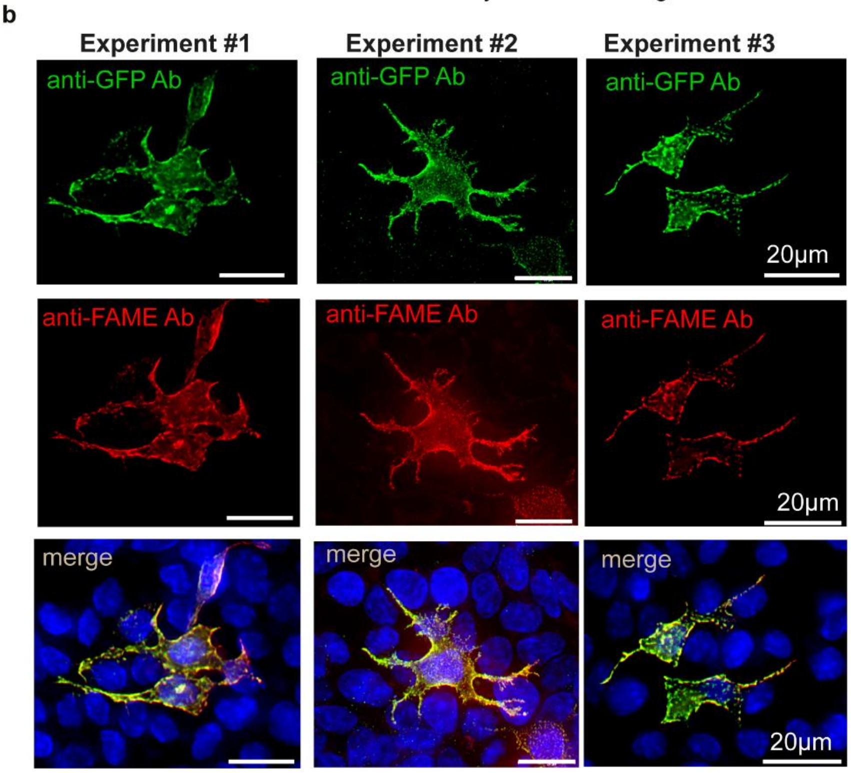

Supplementary Figure 6: FAME antibody validation and staining optimization in cells

(a) Validation of the used mouse monoclonal antibody by immunocytochemistry. FAME fused to the green fluorescent protein EGFP was overexpressed in HEK293T cells. The same cells were stained with the used FAME-specific antibody. Note that only cells expressing FAME-EGFP show a signal in the red antibody-stained channel. There is no background staining observed in HEK293T cells that do not express FAME endogenously. **(b)** as (a) with additional heat-based antigen retrieval. Antigen retrieval leads to a near-perfect co-localisation suggesting increased performance of the used antibody. **(a-b)** Representative images from 3 independent experiments are shown. **(c)** Schematic vector map of the used pEGFP-N1 plasmid with the inserted FAME cDNA. The stop codon was removed to create a fusion protein with EGFP.

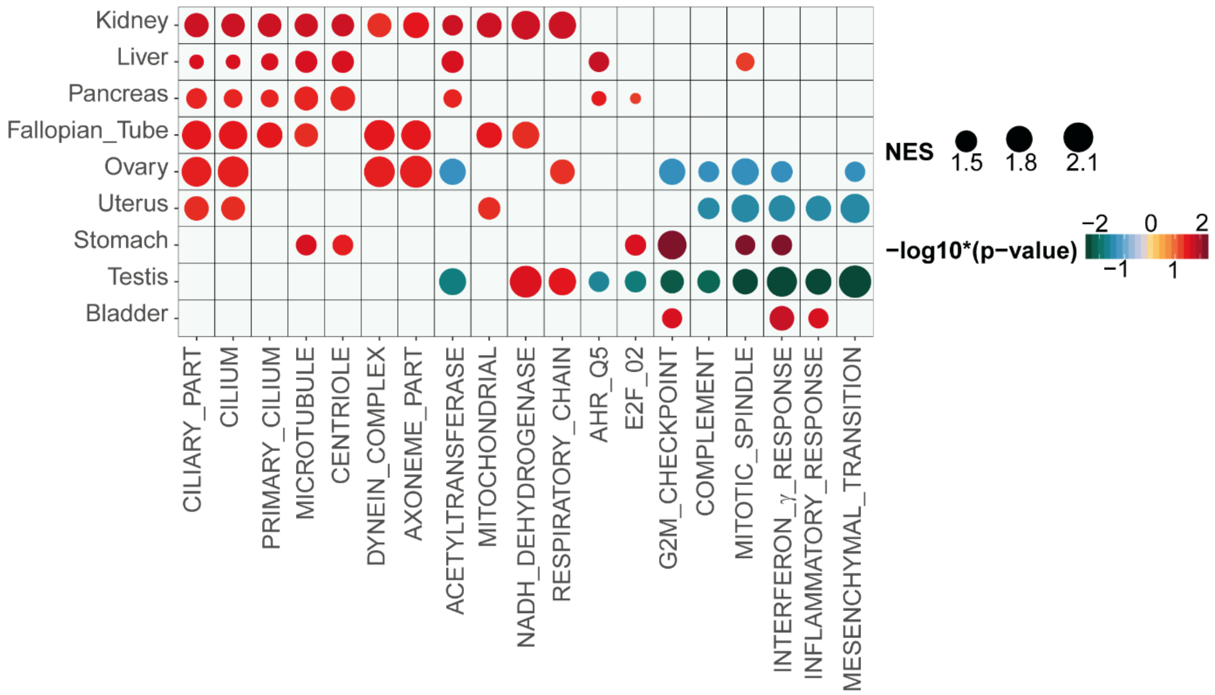

Supplementary Figure 7: Gene correlation enrichment analysis using Genotype-Tissue Expression data

Gene correlation enrichment analysis using Genotype-Tissue Expression data. The human tissues with highest *FAME* expression are ordered in a descending manner.

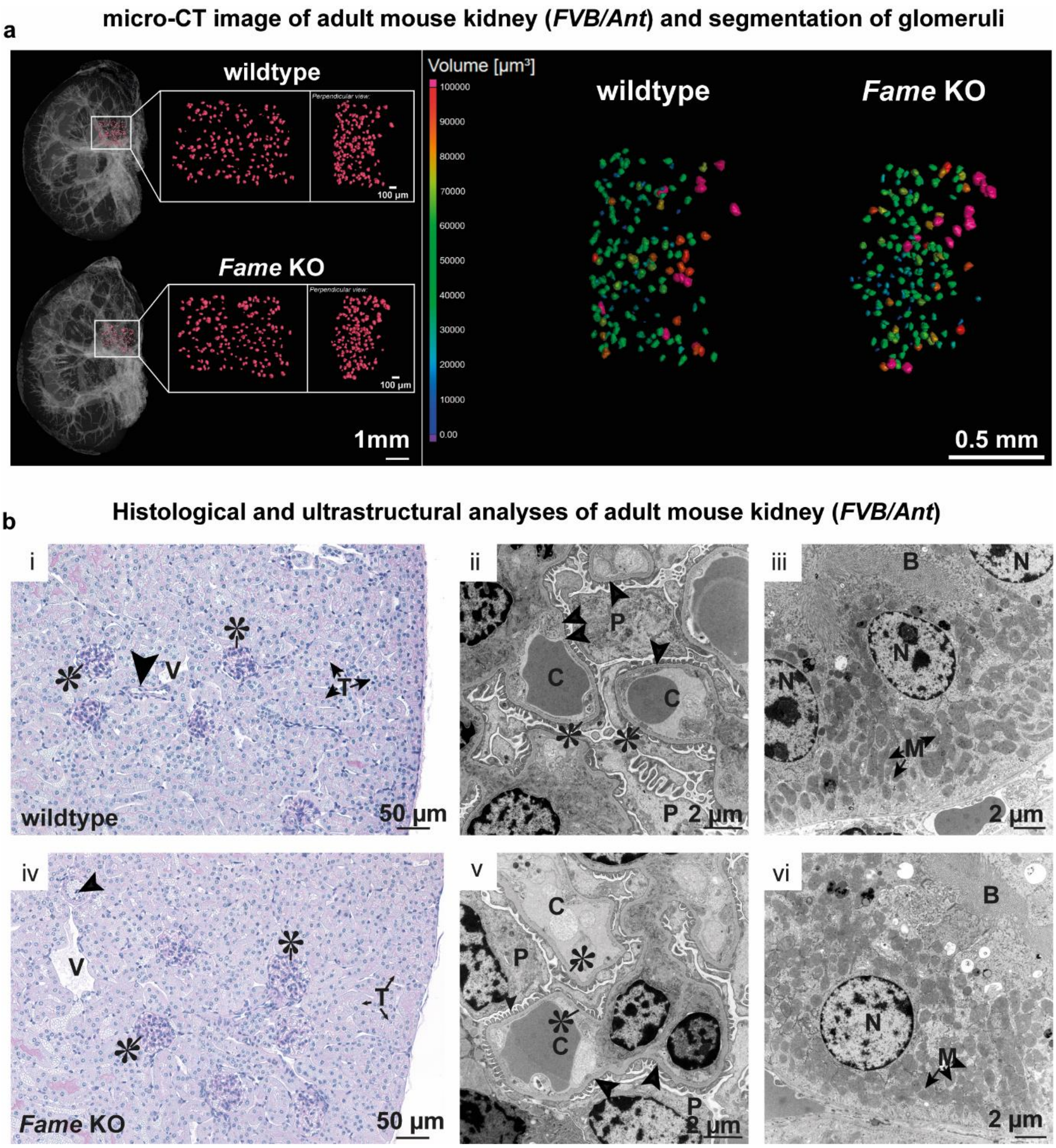

Supplementary Figure 8: Structural analyses of *Fame* KO kidneys

(**a**) Micro-CT images of wild type and *Fame* KO kidneys. Glomeruli were segmented and visualized in dark red. The right panel shows the volume analysis of the segmented glomeruli. No statistically significant differences were observed. **(b)** Histological (i, iv) and ultrastructural analyses (ii, iii, v, vi) show no morphological changes in *Fame* KO mice. **(a-b)** Representative images from 3 independent experiments are shown. **(i, iv)** PAS-stained paraffin sections of wild type and *Fame* KO mice show regularly developed kidneys. (T= tubuli, V= vein, arrow= artery, asterisk= glomeruli) **(ii, v)** Ultrastructural analyses by transmission electron microscopy of glomeruli show intact glomerular filtration barriers with regularly shaped podocyte foot processes (arrows), glomerular basal membrane (asterisks) and thin fenestrated endothelium of glomerular capillaries (C). (P= podocyte body) **(iii, vi)** The tubular cells show typical differentiated morphology with a prominent brush border (B) and several mitochondria (M).

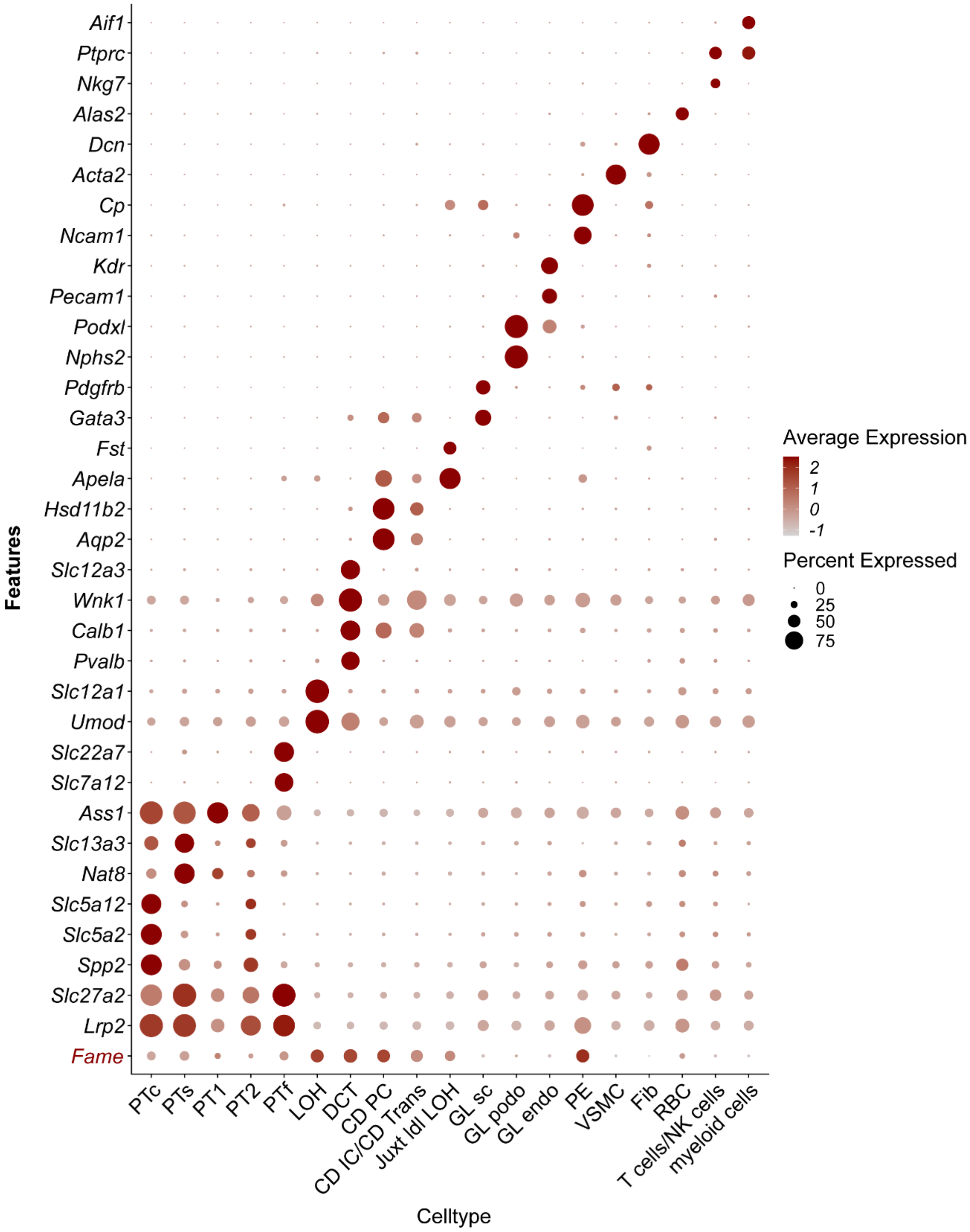
Aif1
Ptprc
Nkg7
Alas2
Dcn
Acta2
Cp
Ncam1
Kdr
Pecam1
Podxl
Nphs2
Pdgfrb
Gata3
Fst
Apela
Hsd11b2
Aqp2
Slc12a3
Wnk1
Calb1
Pvalb
Slc12a1
Umod
Slc22a7
Slc7a12
Ass1
Slc13a3
Nat8
Slc5a12
Slc5a2
Spp2
Slc27a2
Lrp2
Fame
Features
PTc
PTs
PT1
PT2
PTf
LOH
DCT
CD PC
CD IC/CD Trans
Juxt Idl LOH
GL sc
GL podo
GL endo
PE
VSMC
Fib
RBC
T cells/NK cells
myeloid cells
Celltype
Average Expression
2
1
0
-1
Percent Expressed
0
25
50
75

Supplementary Figure 9: Marker genes of cell types found in the adult mouse kidney

Dot plot of marker genes for identification of the cell types corresponding to each cluster in the adult mouse kidney scRNA-seq combined dataset. PTc= proximal convoluted tubules (segment S1), PTs= proximal straight tubule, PTf= female-specific cells from segment S3, PT1= unidentified subcluster of PT, PT2= unidentified subcluster of PT, GL endo= endothelial cells in glomeruli, GL podo= podocytes in glomeruli, GL sc= putative stem cells, LOH= loop of Henle, DCT= distal convoluted tubule, CD PC= collecting duct principal cells, CD IC/CD Trans= collecting duct intercalated cells and transitional cells, VSMC= vascular smooth muscle cells, Juxt ldl LOH= long descending limb of the loop of Henle in juxtamedullary nephrons, PE= parietal epithelium in glomeruli, Fib= fibroblasts, RBC= red blood cells.

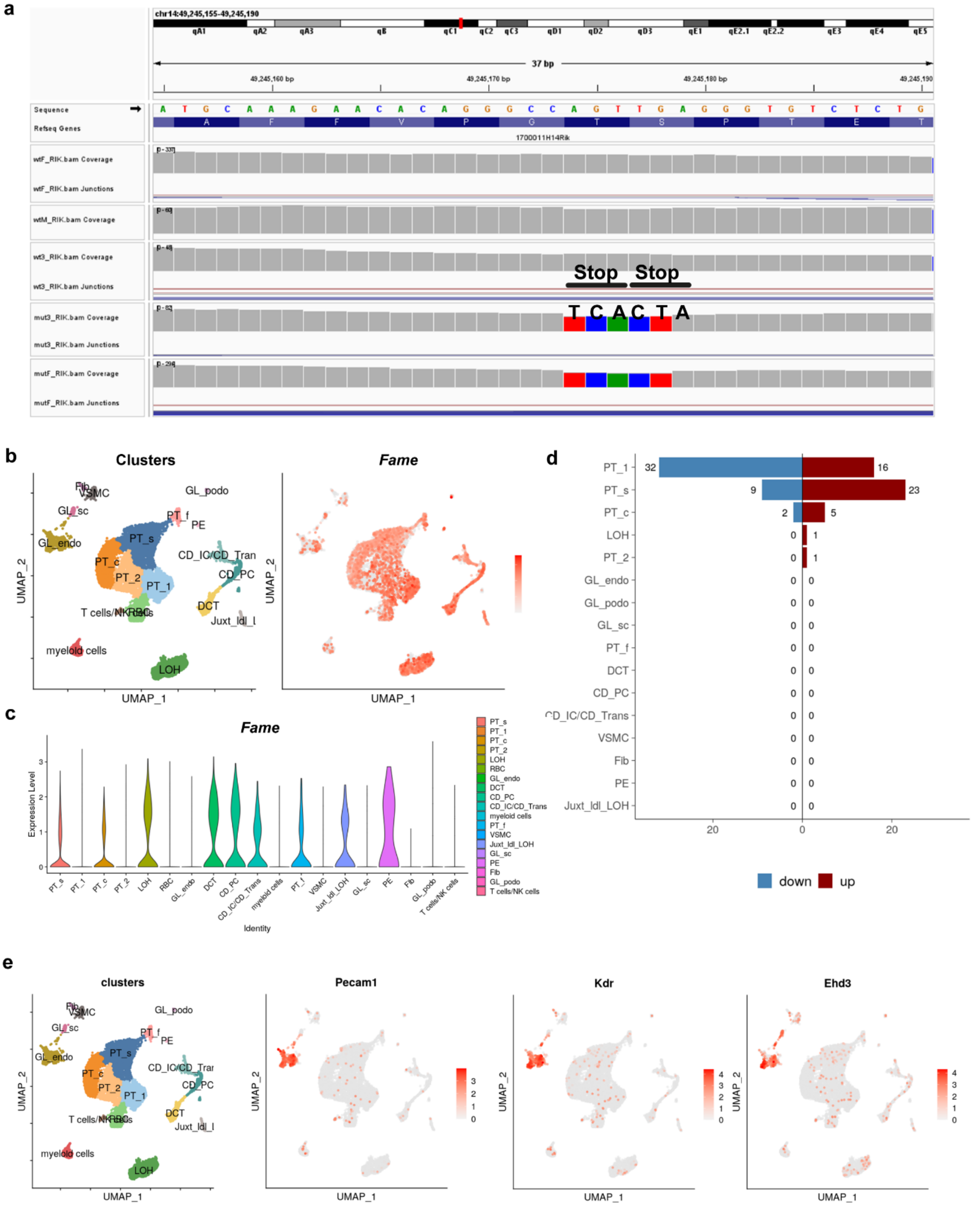

Supplementary Figure 10: Single-cell transcriptomics data of *Fame* KO kidneys

(a) Validation of *Fame* KO mouse (*FVB/Ant* background). Genomic DNA sequences of the indicated locus on chromosome 14 are shown. Note the insertion of a tandem stop codon in *Fame* KO animals. The sequencing reads are visualized by the IGV desktop application (Robinson, Thorvaldsdottir et al. 2011). **(b)** UMAP embedding of single-cell RNA sequencing data and the expression pattern of *Fame* are shown. **(c)** Violin plots of *Fame* expression levels in different cell types/clusters. *Fame* is expressed in all cluster with highest levels in the proximal tubules, collecting duct, loop of Henle, and distal convoluted tubule-associated

clusters. **(d)** Butterfly plot of up- and downregulated genes between wild type and *Fame* KO conditions in different clusters (adjusted p-values <0.01). PT1 contains the most differentially expressed genes between wild type and KO conditions. **(e)** Cluster annotation and expression patterns of *Pecam1*, *Kdr* and the glomerular endothelial cell specific marker *Ehd3* in the GL endo cluster.

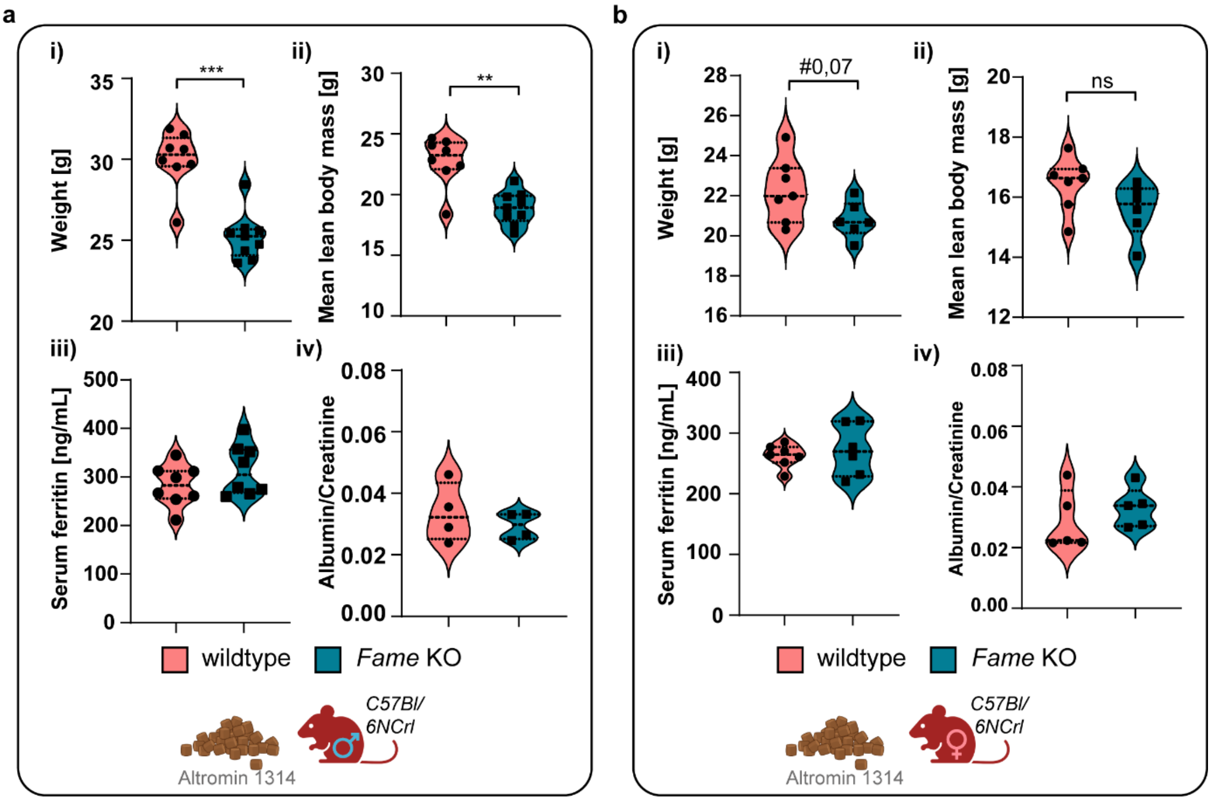

Supplementary Figure 11: Phenotype comparison of *Fame* KO mice (*C57Bl/6NCrl*)

(a-b) Phenotype comparison of the second *Fame* KO mouse model on *C57Bl/6NCrl* genetic background. Serum was collected at sacrificing at the 18-week timepoint. **(a)** 10-week-old male mice on indicated diet. **(i)** Body weight. Mean ± SEM and n: WT (30.01 ± 0.6319 n=8), KO (25.22 ± 0.481 n=9), p-value WT vs KO <0.001 **(ii)** Mean lean body mass. Mean ± SEM and n: WT (22.79 ± 0.7147 n=8), KO (18.90 ± 0.4462 n=9), p-value Mann Whitney test WT vs KO=0.0016 **(iii)** Serum ferritin. Mean ± SEM and n: WT (282.7 ± 14.95 n=8), KO (314.6 ± 18.28 n=8) **(iv)** Urine albumin to creatinine ratio. Urine was collected from 14-week-old mice. WT (0.03362 ± 0.004791 n=4), KO (0.02939 ± 0.002206 n=4) **(b)** 10-week-old female mice on indicated diet. **(i)** Body weight. Mean ± SEM and n: WT (22.27 ± 0.6043 n=7), KO (20.80 ± 0.3696 n=6), p-value WT vs KO= 0.0721 **(ii)** Mean lean body mass. Mean ± SEM and n: WT (16.44 ± 0.3369 n=7), KO (15.58 ± 0.365 n=6) **(iii)** Serum ferritin. Mean ± SEM and n: WT (262.7 ± 7.028 n=7), KO (271.8 ± 17.31 n=6) **(iv)** Urine albumin to creatinine ratio. WT (0.02866 ± 0.004448 n=5), KO (0.03312 ± 0.002937 n=5). Source data are provided as a Source Data file.

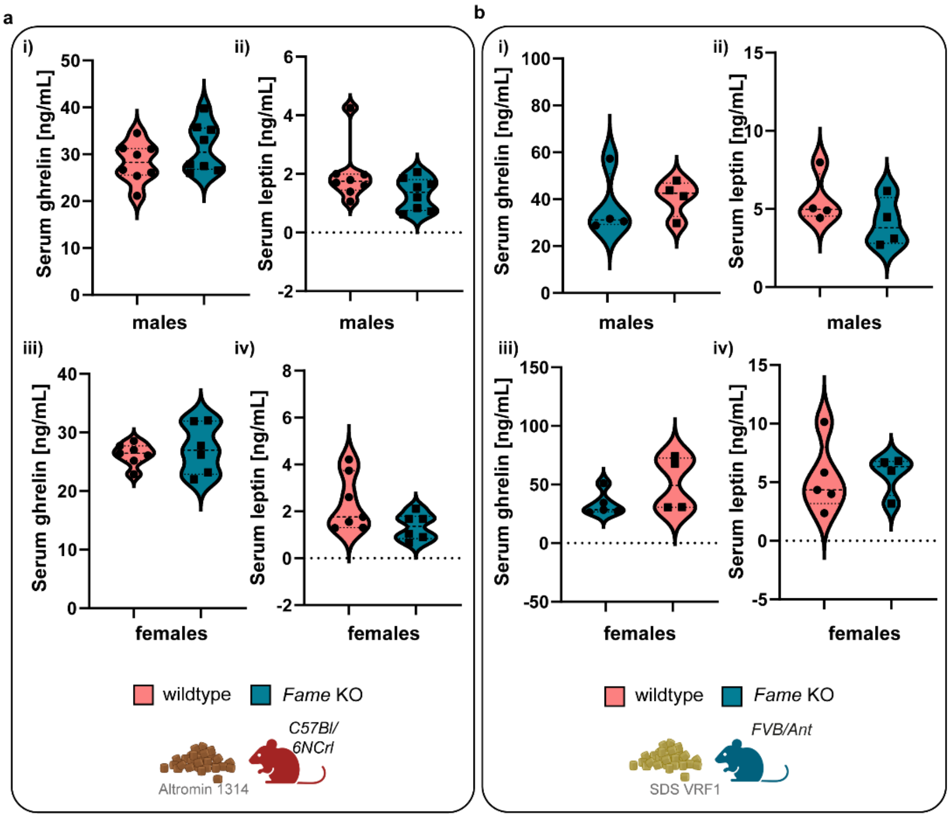

Supplementary Figure 12: Analysis of ghrelin and leptin serum levels

(a-b) Levels of the energy balance influencing hormones ghrelin and leptin compared between *Fame* KO mice on different genetic backgrounds. Serum was collected at sacrificing at the 18-week timepoint. **(a)** Male and female *C57Bl/6NCrl Fame* KO mice. **(i)** Serum ghrelin in male mice. Mean ± SEM and n: WT (28.27 ± 1.495 n=8). KO (31.46 ± 1.827 n=8) **(ii)** Serum leptin in male mice. Mean ± SEM and n: WT (1.975 ± 0.3443 n=8), KO (1.314 ± 0.1929 n=8) **(iii)** Serum ghrelin in female mice. Mean ± SEM and n: WT (26.27 ± 0.7028 n=7), KO (27.18 ± 1.731 n=6) **(iv)** Serum leptin in female mice. Mean ± SEM and n: WT (2.350 ± 0.4543 n=7), KO (1.345 ± 0.2288 n=6) **(b)** Male and female *FVB/Ant* Fame KO mice. **(i)** Serum ghrelin in male mice. Mean ± SEM and n: WT (37.06 ± 6.763 n=4), KO (40.68 ± 3.886 n=4) **(ii)** Serum leptin in male mice. Mean ± SEM and n: WT (5.590 ± 0.8075 n=4), KO (4.113 ± 0.7777 n=4) **(iii)** Serum ghrelin in female mice. Mean ± SEM and n: WT (33.51 ± 4.621 n=5), KO (50.83 ± 11.73 n=4) **(iv)** Serum leptin in female mice. Mean ± SEM and n: WT (5.330 ± 1.324 n=5), KO (5.663 ± 0.8480 n=4). Source data are provided as a Source Data file.

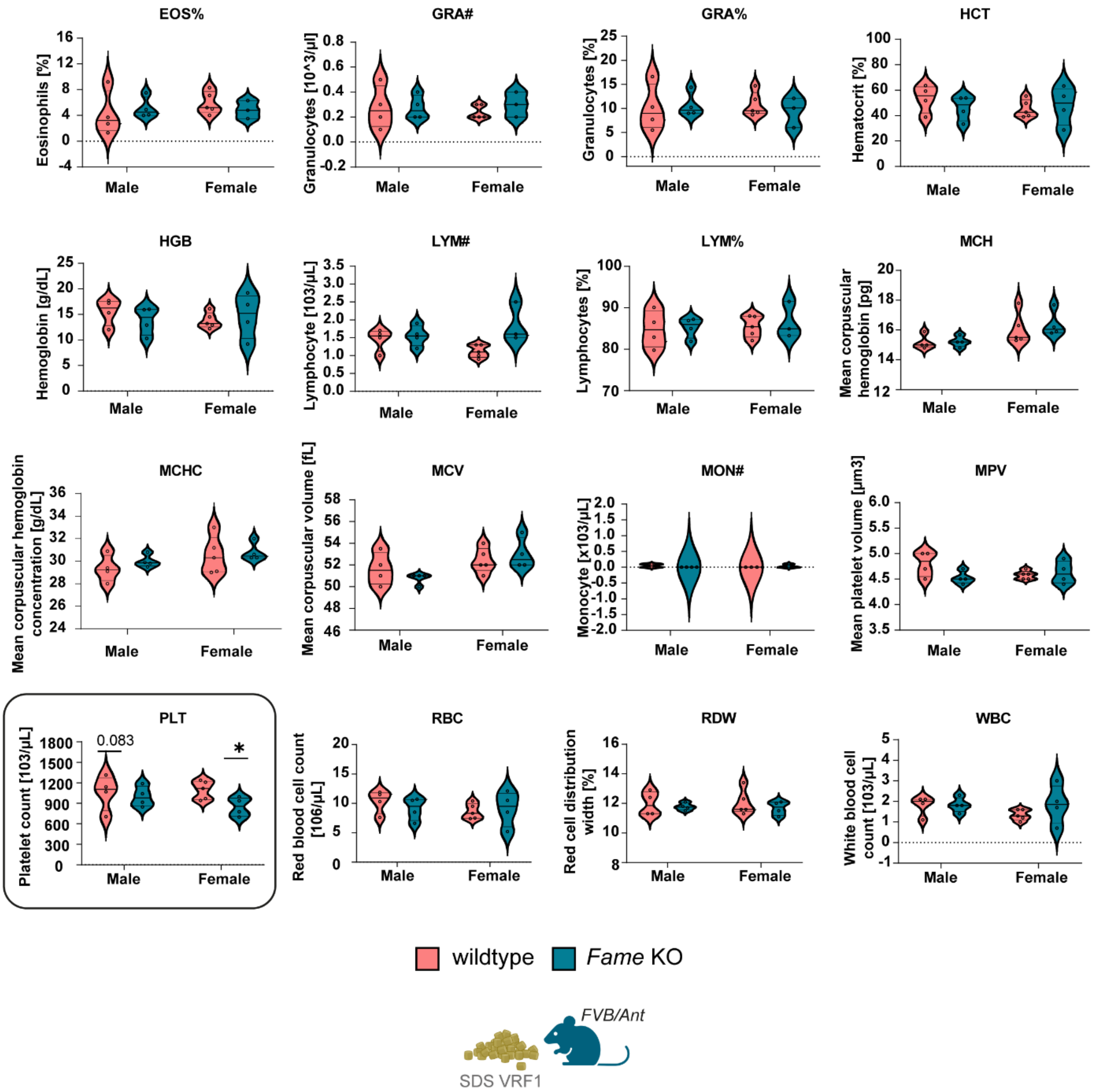

Supplementary Figure 13: Whole blood count of *Fame* KO mice (*FVB/Ant*)

Fame KO mice on an *FVB/Ant* background do not exhibit signs of anaemia and present with a normal blood count except of reduced platelet numbers in females. Platelet count. Mean ± SEM and n: WT male (1057 ± 128.3 n=4), KO male (997.8 ± 74.94 n=4). WT female (1094 ± 60.52 n=5), KO female (845.8 ± 70.39 n=4), p-value female WT vs KO=0.0310. Descriptive statistics of the tested parameters that were not found to be significantly altered can be accessed in Supplementary Data 13. Source data are provided as a Source Data file.

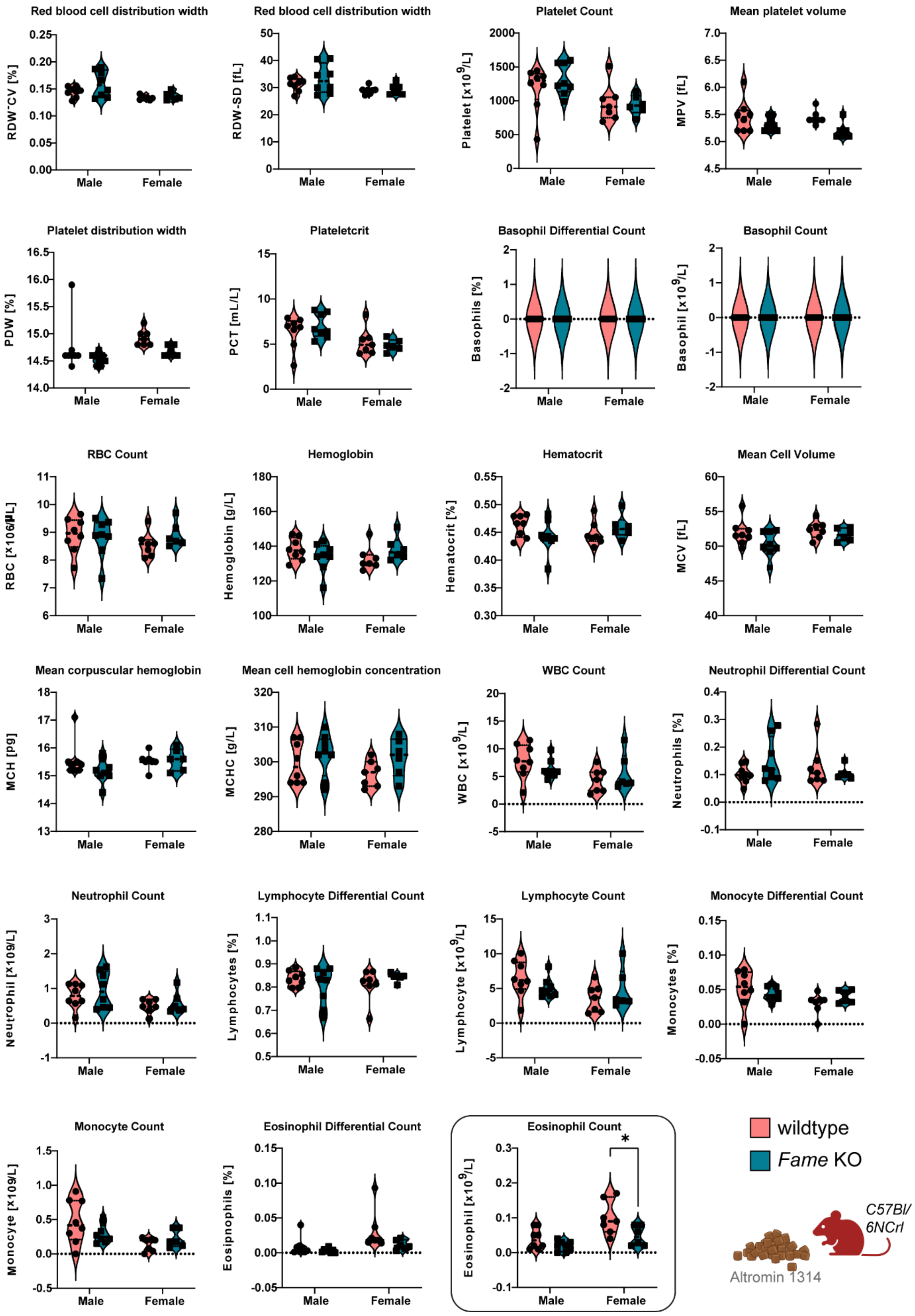

Red blood cell distribution width
RDW-CV [%]
Red blood cell distribution width
RDW-SD [fL]
Platelet Count
Platelet [x10^9/L]
Mean platelet volume
MPV [fL]
Platelet distribution width
PDW [%]
Plateletcrit
PCT [mL/L]
Basophil Differential Count
Basophils [%]
Basophil Count
Basophil [x10^9/L]
RBC Count
Hemoglobin
Hemoglobin [g/L]
Hematocrit
Hematocrit [%]
Mean Cell Volume
MCV [fL]
Mean corpuscular hemoglobin
MCH [pg]
Mean cell hemoglobin concentration
MCHC [g/L]
WBC Count
WBC [x10^9/L]
Neutrophil Differential Count
Neutrophils [%]
Neutrophil Count
Neutrophil [x109/L]
Lymphocyte Differential Count
Lymphocytes [%]
Lymphocyte Count
Lymphocyte [x10^9/L]
Monocyte Differential Count
Monocytes [%]
Monocyte Count
Monocyte [x109/L]
Eosinophil Differential Count
Eosinophils [%]
Eosinophil Count
Eosinophil [x10^9/L]
Male
Female
*
wildtype
Fame KO
C57Bl/
6NCrl
Altromin 1314

Supplementary Figure 14: Whole blood count of *Fame* KO mice (*C57Bl/6Crl*)

Fame KO mice on a *C57Bl/6NCrl* background show a normal blood count except for decreased numbers of eosinophils in female animals. Platelets of knockout animals differ in size from those of wild type animals. Eosinophil count. Mean ± SEM and n: WT male (0.04 ± 0.0104 n=8), KO male (0.01875 ± 0.00515 n=8), WT female (0.100 ± 0.0184 n=7), KO female (0.0483 ± 0.0117 n=6), p-value female WT vs KO= 0.0437, p-value male WT vs KO=0.0874; Mean platelet volume. Mean ± SEM and n: WT male (5.463 ± 0.1068 n=8), KO male (5.325 ± 0.0453 n=8), WT female (5.443 ± 0.0481 n=7), KO female (5.20 ± 0.0633 n=6), p-value female WT vs KO= 0.0227 (Mann-Whitney test); Platelet distribution width. Mean ± SEM and n: WT male (14.75 ± 0.1669 n=8), KO male (14.54 ± 0.0375 n=8), WT female (14.93 ± 0.0565 n=7), KO female (14.68 ± 0.0401 n=6), p-value female WT vs KO=0.0117 (Mann-Whitney test). Descriptive statistics of the tested parameters that were not found to be significantly altered can be accessed in Supplementary Data 13. Source data are provided as a Source Data file.

Lorem ipsum

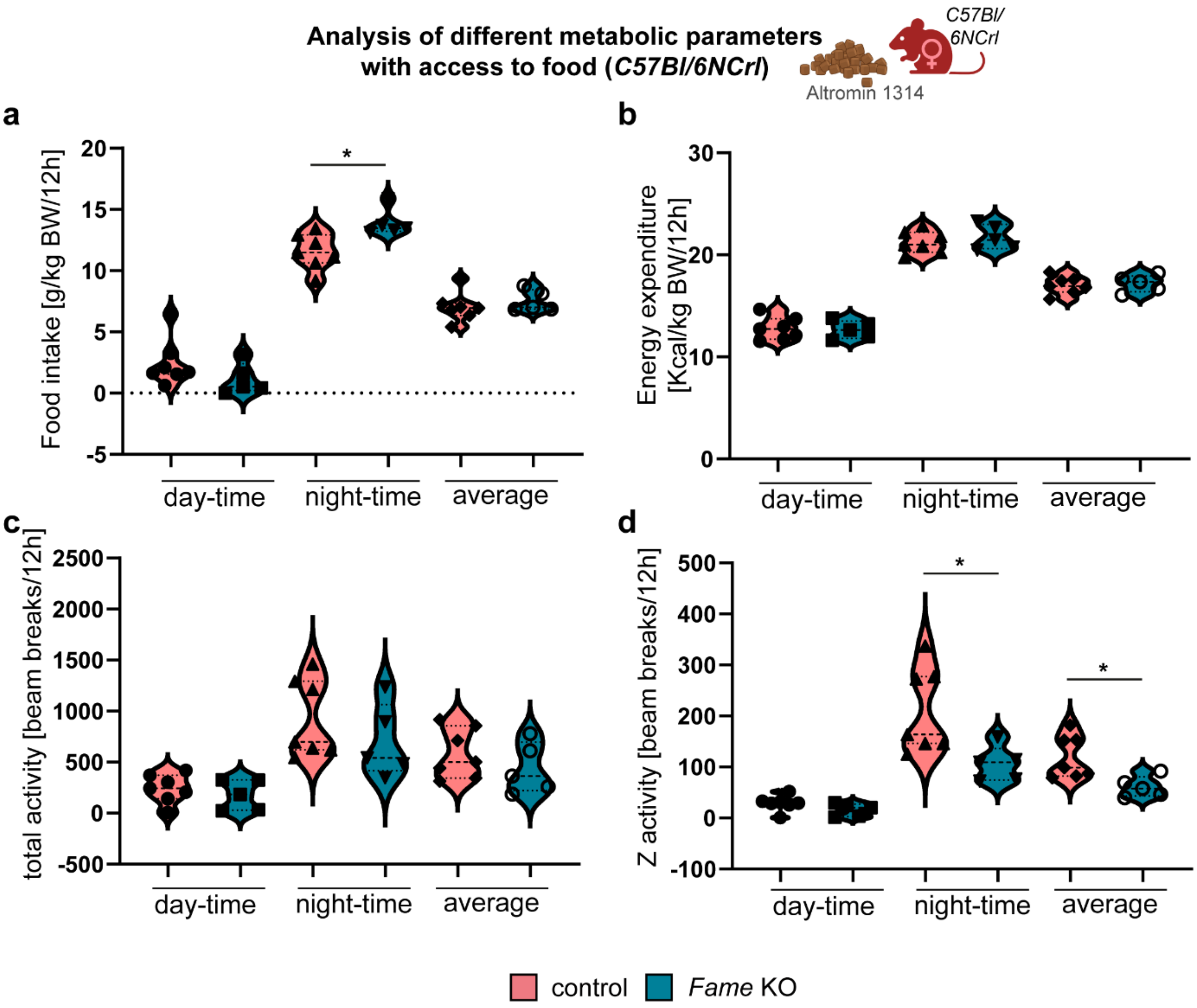

Supplementary Figure 15: Metabolic parameters and activity of female *Fame* KO mice (*C57Bl/6NCrl*)

(**a-d**) Violin plots for metabolic cage experiments with 10-week-old female *C57Bl/6NCrl* wild type and *Fame* KO mice averaged on day-time (6:00-18:00), night-time (18:00-6:00) and average (06:00 – 06:00). (**a**) Food intake of control and *Fame* KO animals normalised to body weight. Female *Fame* KO mice on a *C57Bl/6NCrl* background have a higher food intake at night than wild type counterparts. Day-time WT (2.476 ± 0.7255 n=7) or KO (1.152 ± 0.5717 n=5), night-time WT (11.58 ± 0.5474 n=7) or KO (13.89 ± 0.5058 n=5), average WT (7.029 ± 0.4530 n=7) or KO (7.524 ± 0.3914 n=5), p-value night-time WT vs KO=0.0101 (Mann-Whitney test) **(b)** Energy expenditure of control and *Fame* KO animals normalized to body weight. Day-time WT (12.78 ± 0.4252 n=7) or KO (12.66 ± 0.3955 n=5), night-time WT (21.26 ± 0.4142 n=7) or KO (21.73 ± 0.5472 n=5), average WT (17.02 ± 0.3280 n=7) or KO (17.20 ± 0.3788 n=5). **(c)** Total activity of control and *Fame* KO mice. Day-time WT (242 ± 53.30 n=7) or KO (178.3 ± 66.51 n=5), night-time WT (924.8 ± 144.4 n=7) or KO (700.4 ± 161.3 n=5), average WT (583.4 ± 92.39 n=7) or KO (439.4 ± 111.5 n=5). **(d)** Z-activity of control and *Fame* KO mice. Female *Fame* KO mice show a significant reduction in Z-activity during night-time. Day-time WT (28.61 ± 5.647 n=7) or KO (16.02 ± 5.639 n=5), night-time WT (209.8 ± 31.67 n=7)

or KO (106 ± 15.63 n=5), average WT (119.2 ± 15.85 n=7) or KO (61.02 ± 9.184 n=5), p-value night-time WT vs KO=0.0272, p-value average WT vs KO=0.0175.

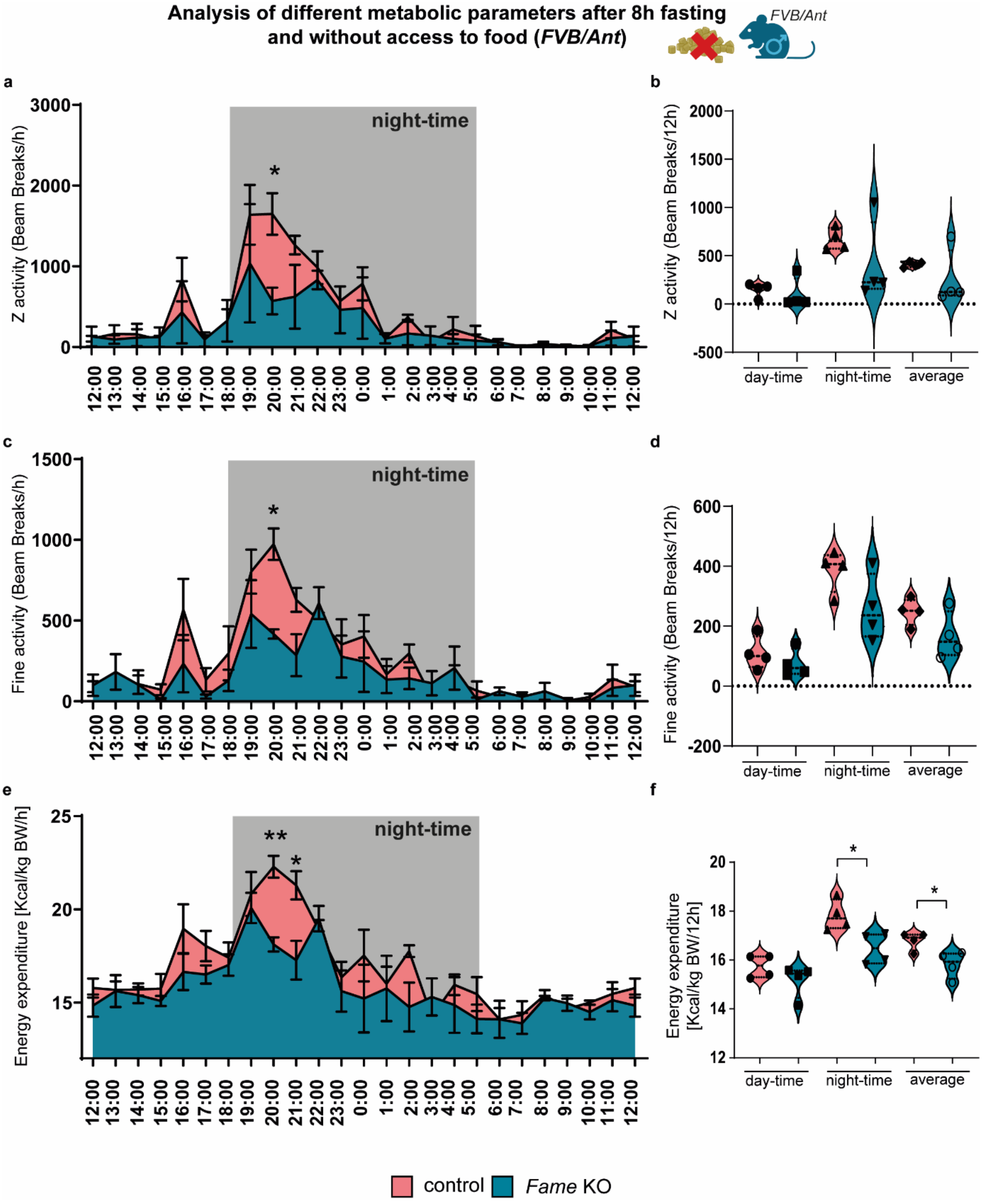

Supplementary Figure 16: Activity profile and energy expenditure of *Fame* KO mice (*FVB/Ant*)

(a-f) Metabolic cage experiment with 75-day old male *FVB/Ant* wild type and *Fame* KO mice after an 8-hour fasting period without further access to food **(a)** Time course analysis of the Z-activity of control and *Fame* KO mice. The Z-activity of *Fame* KO mice is significantly lower at the beginning of the night compared to wild type animals. P-value at 20:00 p=0.0130. **(b)** Violin plots of the Z-activity of control and *Fame* KO mice. Average day-time (6:00-18:00), night-time (18:00-6:00) and average (06:00-06:00) for each animal group is shown. Mean ± SEM and n for each group: day-time WT (148.7 ± 36.04 n=4) or KO (103.7 ± 80.43 n=4), night-time WT (671.8 ± 56.79 n=4) or KO (410.5 ± 215.1 n=4), average WT (410.2 ± 14.84 n=4) or KO (257.1 ± 147.7 n=4) **(c)** Time course analysis of the Fine activity of control and *Fame* KO mice. *Fame* KO animals are less active at the beginning of the night. p-value at 20:00 p=0.0286 (Mann-Whitney test), p-value at 21:00 p=0.0634 **(d)** Violin plots of fine activity. Mean ± SEM and n: day-time WT (110.1 ± 27.9 n=4) or KO (75.34 ± 23.27 n=4), night-time WT (386.1 ± 35.05 n=4) or KO (259 ± 55.67 n=4) p-value WT vs KO=0.1016, average WT (248.1 ± 22.4 n=4) or KO (167.2 ± 39.39 n=4). **(e)** Time course analysis of the energy expenditure of control and *Fame* KO mice normalized to bodyweight. The energy expenditure of *Fame* KO animals is significantly lower than that of wild types at the beginning of the night. p-value at 20:00 p=0.001, p-value at 21:00 p=0.0211 **(f)** Violin plots of energy expenditure. Mean ± SEM and n: day-time WT (15.74 ± 0.2364 n=4) or KO (15.15 ± 0.3387 n=4), night-time WT (17.83 ± 0.313 n=4) or KO (16.47 ± 0.3216 n=4) p-value WT vs KO=0.0228, average WT (16.78 ± 0.1846 n=4) or KO (15.81 ± 0.2752 n=4) p-value WT vs KO=0.0261. (**a, c, e**) Descriptive statistics can be accessed in Supplementary Data 13. Source data are provided as a Source Data file.

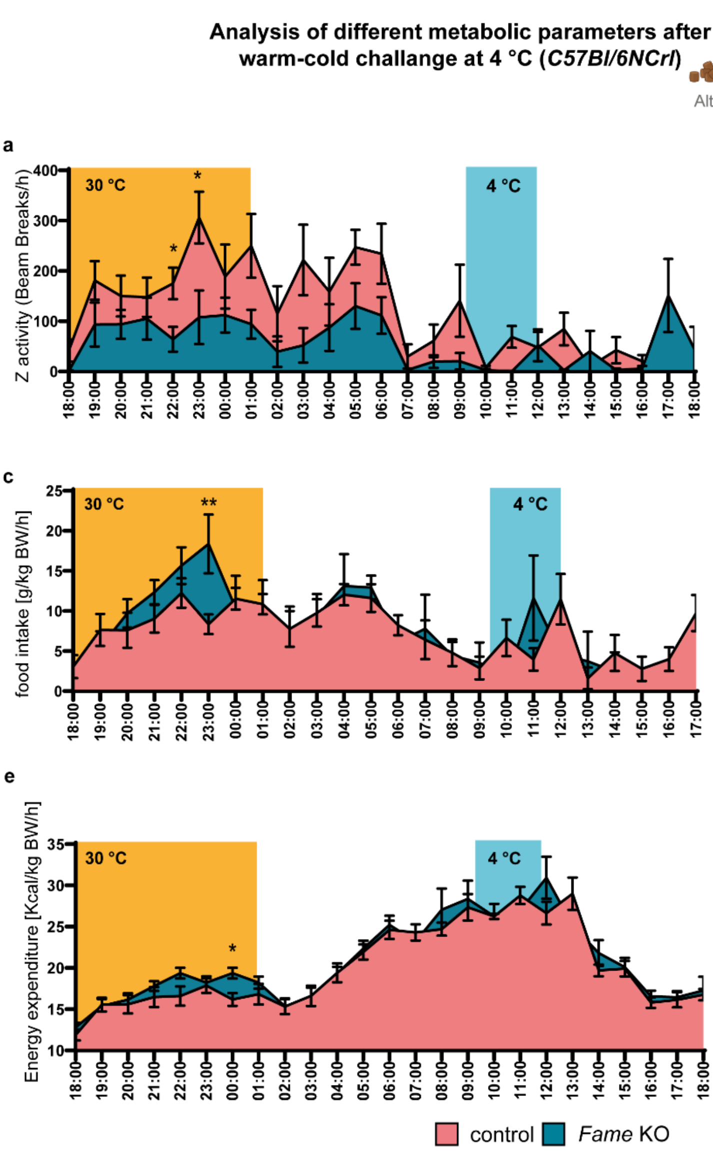

Supplementary Figure 17: Metabolic parameters after warm-cold challenge in female *Fame* KO mice (*C57Bl/6NCrl*)

(a-f) Metabolic cage experiment with 10-week-old female *C57Bl/6NCrl* wild type and *Fame* KO mice challenged with changing ambient temperature (30°C from 18:00 to 01:00 (grey) and 4°C from 09:30 to 12:00 (blue)) **(a)** Time course analysis of the Z-activity of control and *Fame* KO mice. p-value at 22:00=0.0274, p-value at 23:00=0.0259. **(b)** Violin plots of Z-activity. Mean ± SEM and n: day-time WT (63.33 ± 14.14 n=7) or KO (34.42 ± 11.05 n=5), night-time WT (182 ± 23.77 n=7) or KO (81.73 ± 24.72 n=5) p-value WT vs KO=0.0074, average WT (122.7 ± 14.05 n=7) or KO (58.08 ± 10.92 n=5) p-value WT vs KO=0.0070. **(c)** Time course analysis of the food intake of control and *Fame* KO mice normalized to bodyweight. p-value at 23:00=0.0025. **(d)** Violin plots food intake normalized to bodyweight. Mean ± SEM and n: day-time WT (5.60 ± 0.5746 n=7) or KO (4.325 ± 0.8427 n=5), night-time WT (9.287 ± 0.3998 n=7) or KO (10.47 ± 1.305 n=5), average WT (7.443 ± 0.3287 n=7) or KO (7.398 ± 0.7528 n=5). **(e)** Time course analysis of the energy expenditure of control and *Fame* KO mice normalized to bodyweight. p-value at 00:00=0,0147 **(f)** Violin plots of energy expenditure normalized to bodyweight. Mean ± SEM and n: day-time WT (23.57 ± 0.3762 n=7) or KO (24.01 ± 0.6191 n=5), night-time WT (16.81 ± 0.4491 n=7) or KO (17.79 ± 0.3398 n=5), average WT (20.19 ±

0.3147 n=7) or KO (20.90 ± 0.3225 n=5). (**a, c, e**) Descriptive statistics can be accessed in Supplementary Data 13. Source data are provided as a Source Data file.

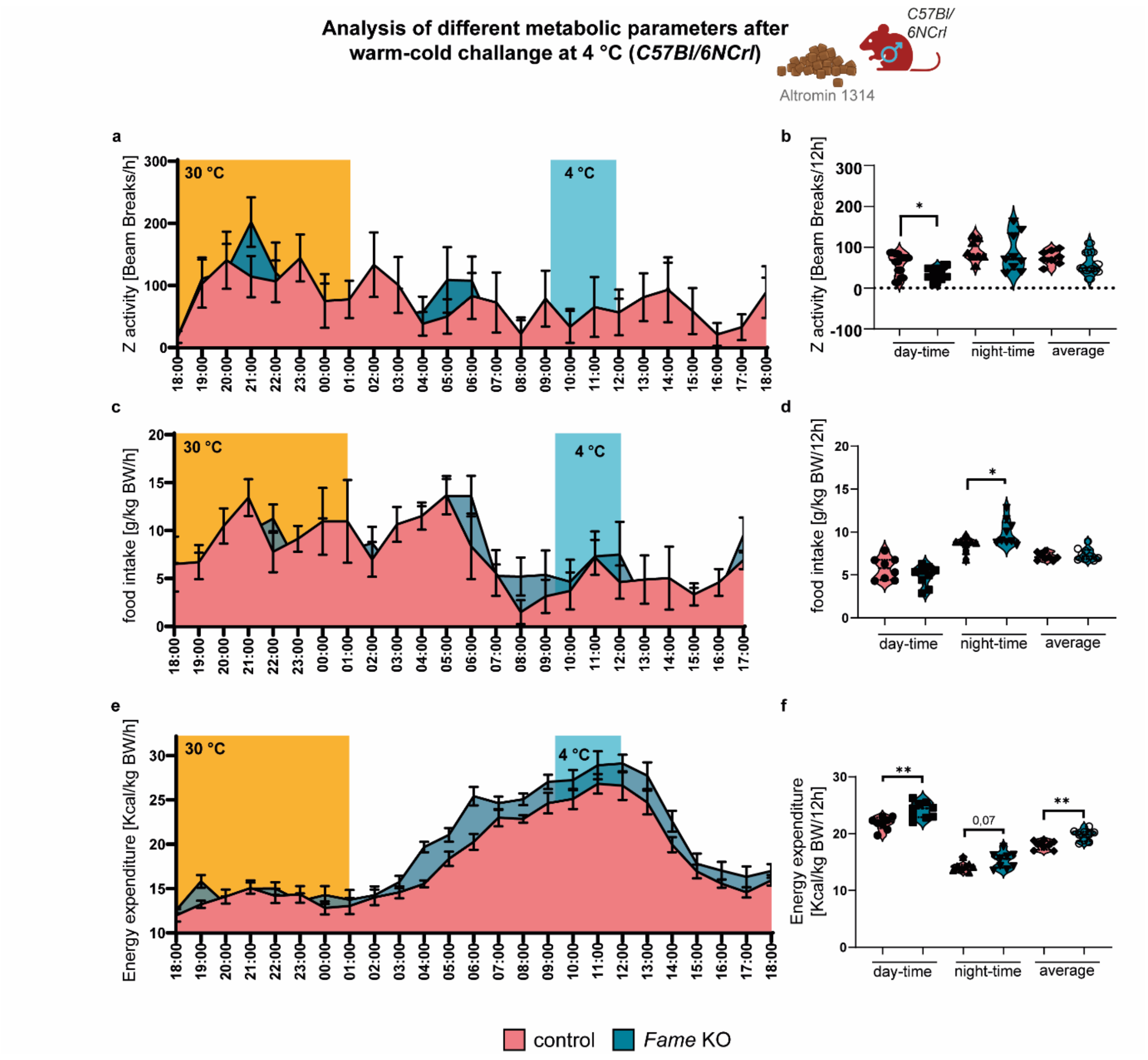

Supplementary Figure 18: Metabolic parameters after warm-cold challenge in male *Fame* KO mice (*C57Bl/6NCrl*)

(a-f) Metabolic cage experiment with 10-week-old male *C57Bl/6NCrl* wild type and *Fame* KO mice challenged with changing ambient temperature (30°C from 18:00 to 01:00 (grey) and 4°C from 09:30 to 12:00 (blue)) **(a)** Time course analysis of the Z-activity of control and *Fame* KO mice with indicated temperature changes. **(b)** Violin plots of Z-activity. *Fame* KO animals have decreased Z-activity compared to wild types during the day-time cold challenge. Mean ± SEM and n: day-time WT (58.23 ± 9.744 n=8) or KO (35.12 ± 5.252 n=9) p-value WT vs KO=0.0478, night-time WT (91.71 ± 9.165 n=8) or KO (85.94 ± 15.61 n=9), average WT (74.97 ± 6.048 n=8) or KO (60.53 ± 9.436 n=9). **(c)** Time course analysis of the food intake of control and *Fame* KO mice normalized to bodyweight. **(d)** Violin plots of food intake normalized to bodyweight. *Fame* KO animals have a significantly higher food intake during the night than wild types. Mean ± SEM and n: day-time WT (5.776 ± 0.4664 n=8) or KO (4.911 ± 0.390 n=9), night-time WT (8.514 ± 0.3021 n=8) or KO (9.901 ± 0.5013 n=9) p-value WT vs KO=0.0366, average WT (7.145 ± 0.1548 n=8) or KO (7.406 ± 0.2430 n=9). **(e)** Time course analysis of the energy expenditure of control and *Fame* KO mice normalized to bodyweight. p-value at

04:00=0.0001, p-value at 05:00=0.0298, p-value at 06:00=0.0037 (Mann-Whitney test) **(f)** Violin plots of energy expenditure normalized to bodyweight. The energy expenditure of *Fame* KO animals was significantly higher than that of controls once the ambient temperature was changed. Mean ± SEM and n: day-time WT (21.82 ± 0.4023 n=8) or KO (24.12 ± 0.4749 n=9) p-value WT vs KO=0.0024, night-time WT (14.18 ± 0.2891 n=8) or KO (15.28 ± 0.4705 n=9) p-value WT vs KO=0.0722, average WT (18.00 ± 0.2673 n=8) or KO (19.70 ± 0.3323 n=9) p-value WT vs KO=0.0014. (**a, c, e**) Descriptive statistics can be accessed in Supplementary Data 13. Source data are provided as a Source Data file.

Genome-Wide Association Study of *FAME*

Phenotype tested in Study	Phenotype ontology annotation	Total p-values in Study	Study	Reference
Age-related macular degeneration	Macular Degeneration	427,832	GWAS of age-related macular degeneration (HGVST4)	(Klein et al., 2005)
Body mass index (HGVPM1111)	Body Mass Index	2,471,541	GWAS of body mass index (HGVST640)	(Hindorff et al., 2009; Speliotes et al., 2010)
Diabetic nephropathy in type I diabetes (HGVPM1531)	Diabetic Nephropathies	706,749	Genetics of kidneys in diabetes (GoKinD) (HGVST890)	(Pezzolesi et al., 2010)
Fasting glucose-related: homeostatic model assessment of beta-cell function (HGVPM827)	Insulin-Secreting Cells	2,456,942	GWAS of glycemic traits (HGVST463)	(Dupuis et al., 2010; Hindorff et al., 2009)
Fasting insulin-related: fasting insulin (HGVPM822)	Insulin	2,461,093	GWAS of glycemic traits (HGVST463)	(Dupuis et al., 2010; Hindorff et al., 2009)
Fasting insulin-related: homeostatic model assessment of insulin resistance (HGVPM826)	Insulin Resistance	2,458,062	GWAS of glycemic traits (HGVST463)	(Dupuis et al., 2010; Hindorff et al., 2009)
Type II diabetes (HGVPM5)	Diabetes Mellitus, Type 2	772,55	GWAS of type II diabetes mellitus (HGVST3)	(Diabetes Genetics Initiative of Broad Institute of et al., 2007)

Supplementary Figure 19: Genome-wide association study of *FAME*

Genome-Wide Association Studies of *FAME* using gwascentral.org

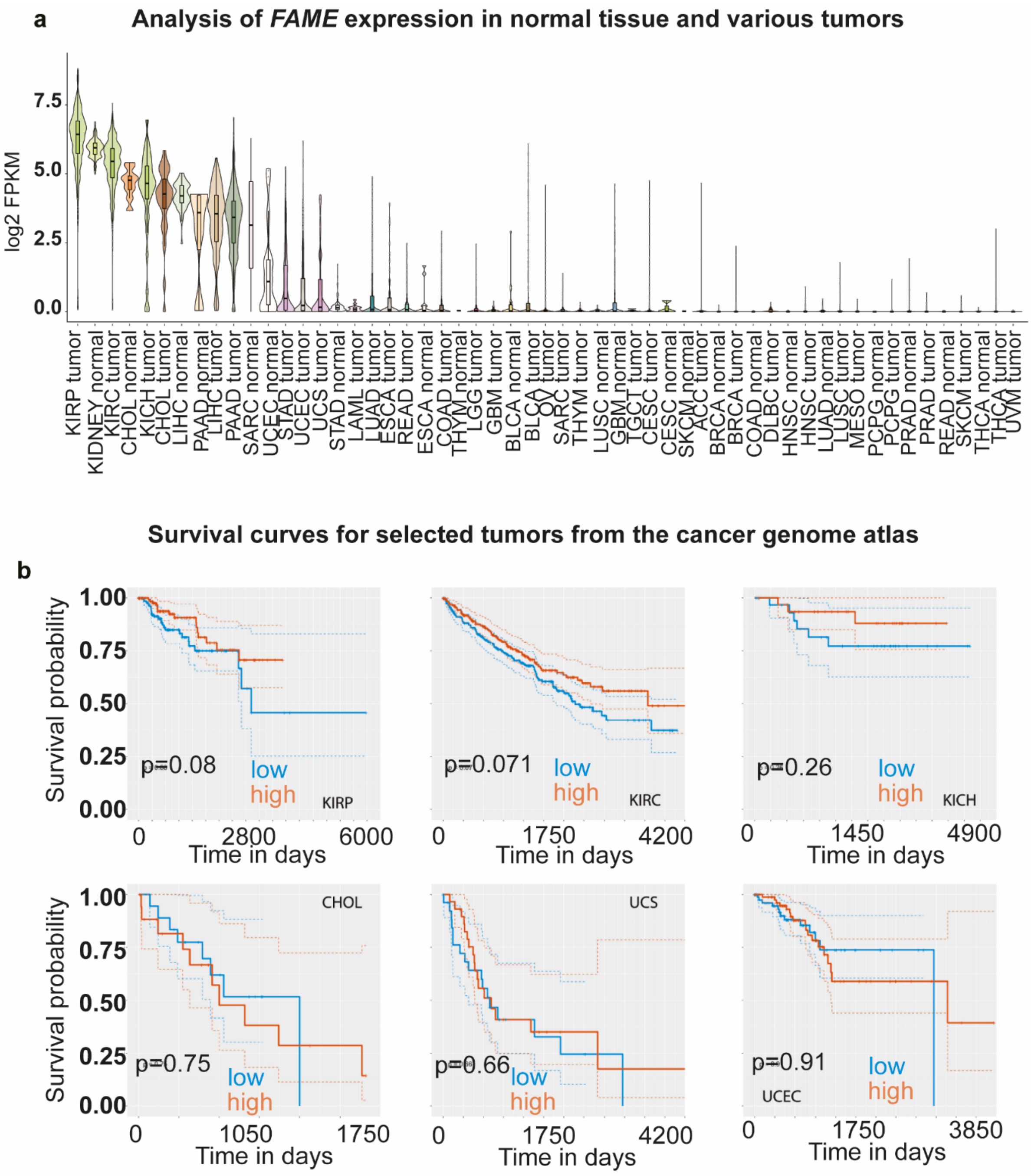

c

	average ct value		
HEK293T	*FAME*	*GAPDH*	ΔCt
WT 1	36,70	20,68	1,51E-05
WT 2	37,40	22,05	2,38E-05
WT 3	37,31	20,30	7,59E-06
WT 4	37,48	20,27	6,62E-06
WT 5	37,22	20,69	1,06E-05

Supplementary Figure 20: *FAME* expression and survival probability in tumors

(a) Analysis of *FAME* expression in various tumors and healthy tissue using RNAseq data from The Cancer Genome Atlas (TCGA) Program **(b)** Survival curves for selected tumors from

TCGA with detectable *FAME* expression. Samples are binned into high (orange) and low (blue) *FAME* expression. KIRP = Kidney renal papillary cell carcinoma, KIRC = Clear cell renal cell carcinoma, KICH = Kidney Chromophobe, CHOL = Cholangiocarcinoma, UCS = Uterine carcinosarcoma, UCEC = Uterine Corpus Endometrial Carcinoma. **(c)** Table highlighting insignificant background levels of *FAME* expression in HEK293T cells measured by qPCR. Averaged values of technical triplicates per analysed HEK293T clone are shown.

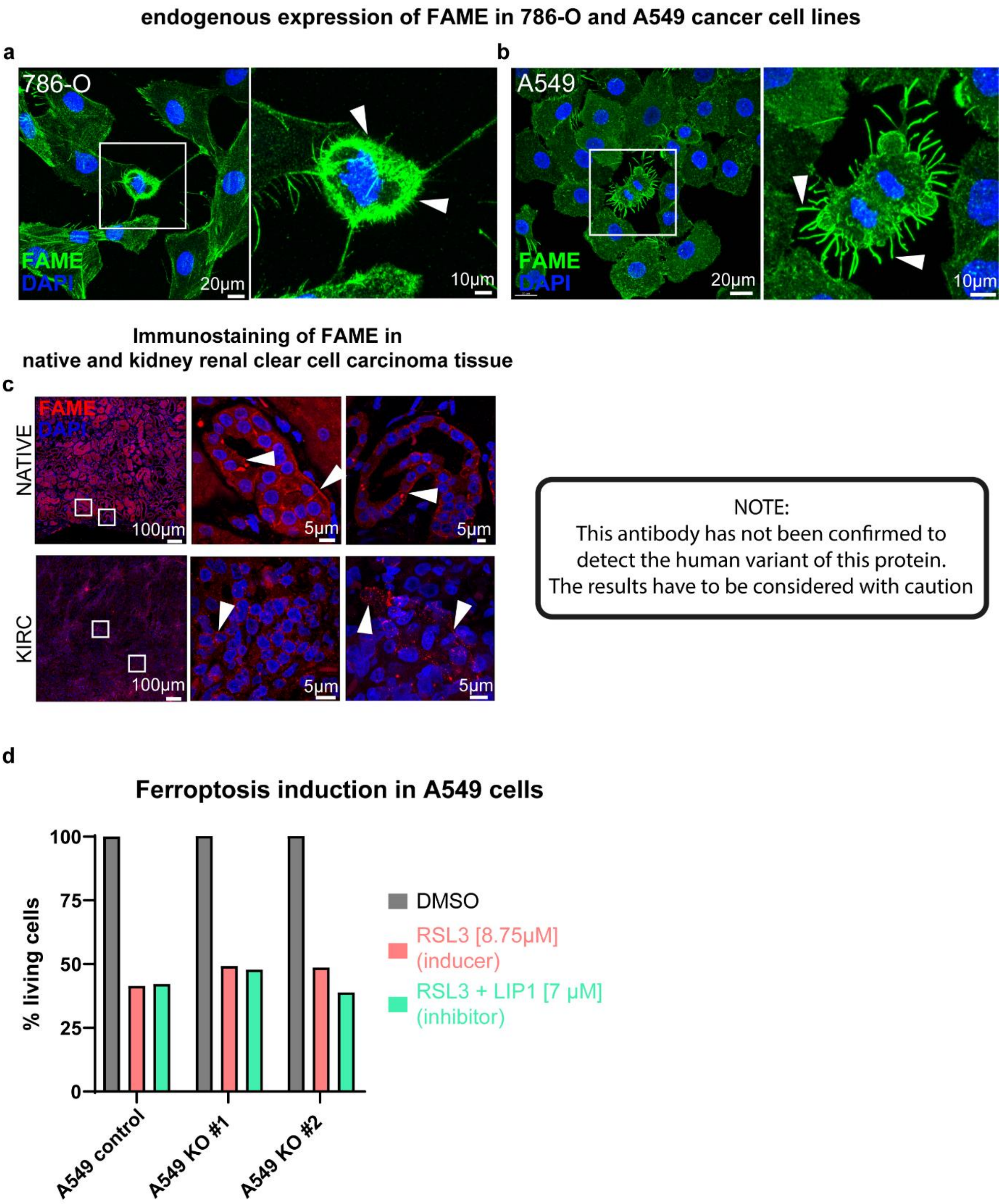

Supplementary Figure 21: Endogenous FAME in cancer tissues and ferroptosis susceptibility

(a) Immunofluorescence staining of endogenous FAME in the human kidney carcinoma cell line 786-O. A mitotic cell is magnified (white box) with arrows pointing towards high FAME levels near the plasma membrane. **(b)** The human lung adenocarcinoma cell line A549 is stained as in (a). **(c)** FAME stainings of kidney renal clear cell carcinoma (KIRC) and healthy control tissue are shown. The white arrows highlight membranous expression (upper panel) as well as vesicular expression (lower panel). **(a-c)** Representative images from 3

independent stainings are shown. **(d)** Representative analysis of the ferroptosis susceptibility of *FAME* KO cells. Two A549 *FAME* KO and a WT control clone were treated with the ferroptosis inducer RSL3 [8,75 µM]. No difference was observed amongst the clones. Of note, the addition of the ferroptosis inhibitor Liproxstatin-1 (LIP1) did not rescue RSL3-induced cell death. Source data are provided as a Source Data file.

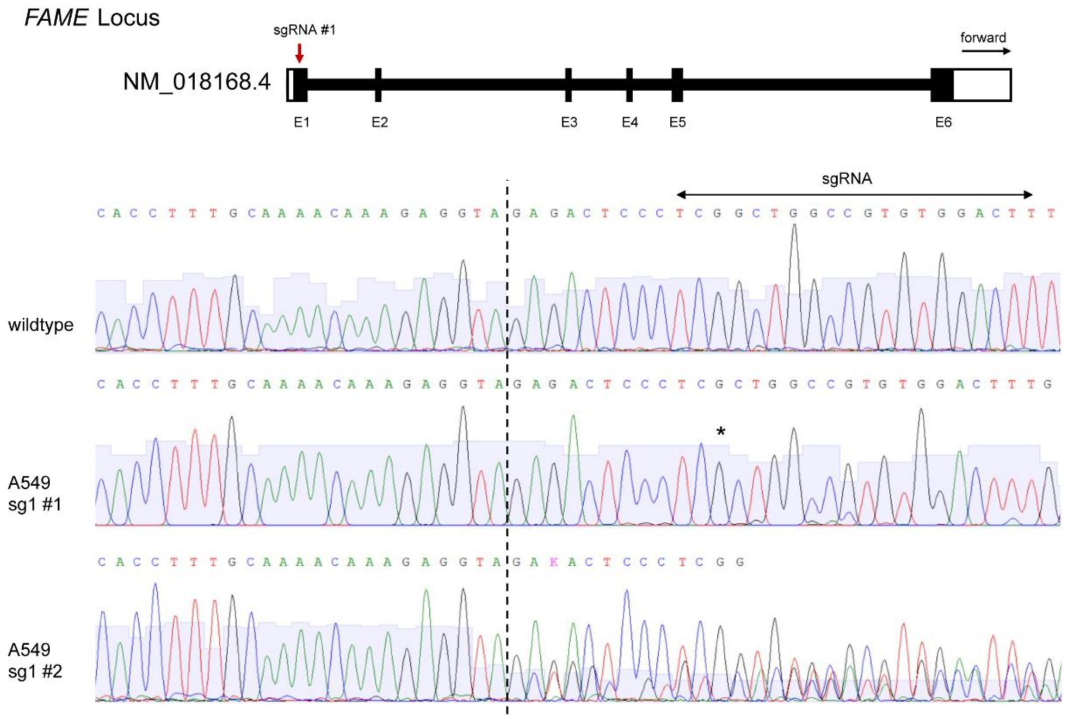

Supplementary Figure 22: *FAME* KO validation by genomic DNA sequencing of A549 cells

FAME KO validation in human A549 cells by genomic DNA sequencing. The FAME locus on chromosome 14 with exons (E) is schematically depicted (top). The target position of the single-guide RNA is shown (red arrow). The used NCBI Reference Sequence is indicated. Sanger sequencing chromatograms of the indicated genomic regions are shown. The 20-nucleotide stretch corresponding to the sgRNA is marked with a black arrow. Editing by Cas9 took place in the expected region, resulting in a homozygous single nucleotide insertion (asterisk) in A549 KO clone #1 and heterozygous editing in clone #2. The dotted line indicates the start of changed positions in the sequence alignments.

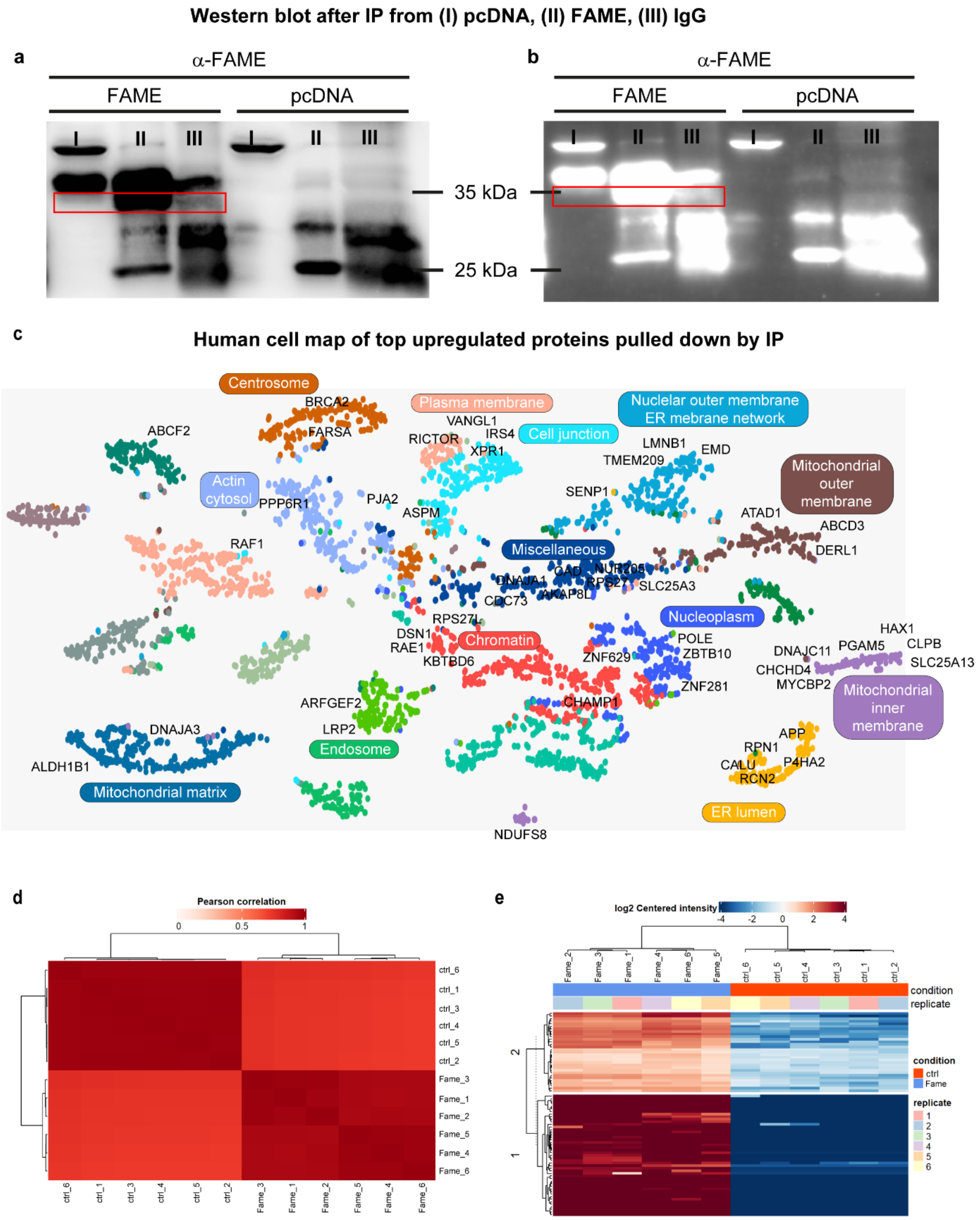

Supplementary Figure 23: Validation and cell-localization representation of IP-MS interactomics approach

(a) Validation of the immunoprecipitation (IP) approach using the mouse monoclonal anti-Fame antibody. FAME was overexpressed and enriched after lysis with the anti-FAME antibody. The empty vector was included as control. A FAME-specific band (predicted 34.69 kDa) is only observed in the FAME overexpressed condition, detected with the anti-FAME antibody on western blot. **(b)** Inverted colours of the western blot from (a). **(a-b)** Data from a single experiment is shown. **(c)** Interaction partners of FAME identified by IP-mass

spectrometry (MS) are visualized in a human cell map of various organelles and cell compartments. **(d)** Heatmap visualizing Pearson correlations between replicates of the IP-MS show a high degree of consistency amongst the control and FAME overexpression conditions. **(e)** Heatmap of the log2 Centred Intensity shows strong upregulation of proteins in the Fame overexpression condition.

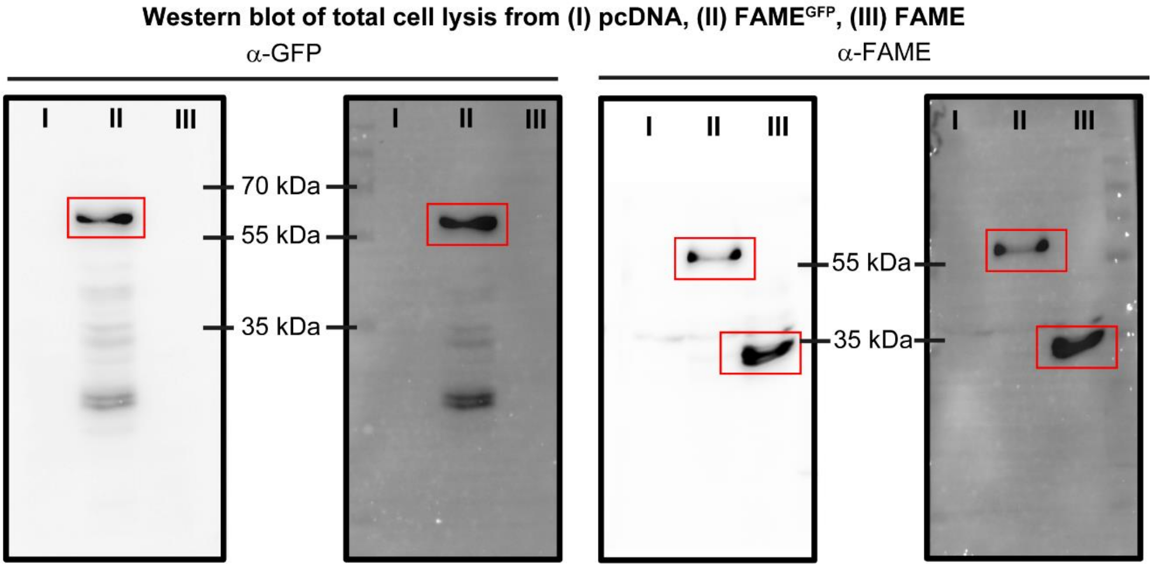

Supplementary Figure 24: Western blot-based antibody validation of overexpressed *FAME*

Overexpression and subsequent western blot analysis of an empty pcDNA3.1 vector (I), pcDNA3.1-FAME-EGFP fusion protein (II) and pcDNA3.1-FAME (III) plasmids in HEK293T cells. Lane II shows a band corresponding to the fusion protein (predicted molecular mass of Fame 34.69 kDa and EGFP 27kDa). Lane III shows a specific band at the expected height for overexpressed FAME only detectable with the anti-FAME antibody. Data from a single experiment is shown.

Supplementary References

Genomes Project, C., A. Auton, L. D. Brooks, R. M. Durbin, E. P. Garrison, H. M. Kang, J. O. Korbel, J. L. Marchini, S. McCarthy, G. A. McVean and G. R. Abecasis (2015). "A global reference for human genetic variation." Nature **526**(7571): 68-74.

Machiela, M. J. and S. J. Chanock (2015). "LDlink: a web-based application for exploring population-specific haplotype structure and linking correlated alleles of possible functional variants." Bioinformatics **31**(21): 3555-3557.

Robinson, J. T., H. Thorvaldsdottir, W. Winckler, M. Guttman, E. S. Lander, G. Getz and J. P. Mesirov (2011). "Integrative genomics viewer." Nat Biotechnol **29**(1): 24-26.

Soldatov, R., M. Kaucka, M. E. Kastriti, J. Petersen, T. Chontorotzea, L. Englmaier, N. Akkuratova, Y. Yang, M. Haring, V. Dyachuk, C. Bock, M. Farlik, M. L. Piacentino, F. Boismoreau, M. M. Hilscher, C. Yokota, X. Qian, M. Nilsson, M. E. Bronner, L. Croci, W. Y. Hsiao, D. A. Guertin, J. F. Brunet, G. G. Consalez, P. Ernfors, K. Fried, P. V. Kharchenko and I. Adameyko (2019). "Spatiotemporal structure of cell fate decisions in murine neural crest." Science **364**(6444).
