# Supplementary material for: A previously uncharacterized Factor Associated with Metabolism and Energy (FAME/C14orf105/CCDC198/1700011H14Rik) is related to evolutionary adaptation, energy balance, and kidney physiology
Source: Nat Commun. 2023 May 29;14:3092. doi: 10.1038/s41467-023-38663-7 (PMC10226981; doi:10.1038/s41467-023-38663-7)
Supplement: Supplementary file 3 — Description of Additional Supplementary Files [file 41467_2023_38663_MOESM3_ESM.pdf]

Description of Additional Supplementary Information

Supplementary Data 1: List of used amniote organisms with their NCBI Genome ID.

Supplementary Data 2: Established pairs within each group and median dS for each pair.

Supplementary Data 3: Identified proteins in reptiles and birds with significantly differing dN/dS signatures

Supplementary Data 4: Most flexible reptile protein sequences after bidirectional analysis

Supplementary Data 5: Habitat matrix of different animals

Supplementary Data 6: Significance of domain overlap by selected region related to PanTHERIA (Precip_Mean_mm)

Supplementary Data 7: gProfiler detailed data

Supplementary Data 8: DEP_processing_BH

Supplementary Data 9: p-values of significantly differentially expressed genes in PT1 cell type

Supplementary Data 10: Comparison of sex-specific genes in proximal tubule found in PMID: 31689386

Supplementary Data 11: Metabolic cage RAW Data for *FVB/Ant* and after fasting

Supplementary Data 12: Metabolic cage RAW Data for *C57BL/6NCRLCrl* and after warm/cold challenge

Supplementary Data 13: Descriptive statistics

Supplementary Movie 1: Time lapse videos of HEK293T cells overexpressing EGFP-tagged FAME.

1) A single cell at high magnification is shown. Note the cytoplasmic, vesicular protein localization and fast trafficking and fusion of FAME+ vesicles.

2) A cluster of cells at lower magnification is shown. FAME-EGFP is prominently located in the plasma membrane. Note the sharing or fusing of membranes leading to FAME-EGFP transfer to another cell, as can be seen by green fluorescence signal in the membrane of a GFP- cell (black arrow).
