# Supplementary material for: A previously uncharacterized Factor Associated with Metabolism and Energy (FAME/C14orf105/CCDC198/1700011H14Rik) is related to evolutionary adaptation, energy balance, and kidney physiology
Source: Nat Commun. 2023 May 29;14:3092. doi: 10.1038/s41467-023-38663-7 (PMC10226981; doi:10.1038/s41467-023-38663-7)
Supplement: Supplementary file 10 — Reporting Summary [file 41467_2023_38663_MOESM10_ESM.pdf]

nature portfolio

Corresponding author(s): Igor Adameyko and Julian Petersen

Last updated by author(s): Apr 21, 2023

Reporting Summary

Nature Portfolio wishes to improve the reproducibility of the work that we publish. This form provides structure for consistency and transparency in reporting. For further information on Nature Portfolio policies, see our Editorial Policies and the Editorial Policy Checklist.

Statistics

For all statistical analyses, confirm that the following items are present in the figure legend, table legend, main text, or Methods section.

n/a	Confirmed	
☐	☒	The exact sample size (*n*) for each experimental group/condition, given as a discrete number and unit of measurement
☐	☒	A statement on whether measurements were taken from distinct samples or whether the same sample was measured repeatedly
☐	☒	The statistical test(s) used AND whether they are one- or two-sided<br>*Only common tests should be described solely by name; describe more complex techniques in the Methods section.*
☒	☐	A description of all covariates tested
☐	☒	A description of any assumptions or corrections, such as tests of normality and adjustment for multiple comparisons
☐	☒	A full description of the statistical parameters including central tendency (e.g. means) or other basic estimates (e.g. regression coefficient) AND variation (e.g. standard deviation) or associated estimates of uncertainty (e.g. confidence intervals)
☐	☒	For null hypothesis testing, the test statistic (e.g. *F*, *t*, *r*) with confidence intervals, effect sizes, degrees of freedom and *P* value noted<br>*Give P values as exact values whenever suitable.*
☒	☐	For Bayesian analysis, information on the choice of priors and Markov chain Monte Carlo settings
☒	☐	For hierarchical and complex designs, identification of the appropriate level for tests and full reporting of outcomes
☒	☐	Estimates of effect sizes (e.g. Cohen's *d*, Pearson's *r*), indicating how they were calculated

Our web collection on statistics for biologists contains articles on many of the points above.

Software and code

Policy information about availability of computer code

Data collection

- Incucyte experiments were performed using Essen Bioscience/Sartorius - Software Incucyte 2021C

- For IP data evaluation, the MaxQuant software (2.0.1.0) with inbuild Andromeda search engine was used

- A PhenoMaster (TSE Systems) system was used for the indirect calorimetry. The software used in the PhenoMaster PC was TSE PhenoMaster v.7.1.2.

- Raw single cell sequencing files were processed, mapped, and counted to the Cell Ranger mm10-2020-A genome and its corresponding annotation by Cell Ranger version 4.0.0. The output count matrices for each sample were further processed with the Seurat package pipeline (v.3.2.2.9001)

- Microscopy to capture fluorescence after immunohistochemistry staining using antibodies was performed using a Zeiss LSM880 Airyscan or LEICA THUNDER System with 10, 20, 40 and 63x objectives.

- Quantitative expression analysis was performed using the QuantStudio 5 Real-Time PCR Instrument

- Micro-computed tomography
The micro-CT scanning of the pups was performed using the laboratory system GE phoenix v|tome|x L 240 (GE Sensing & Inspection Technologies GmbH, Germany), equipped with a 180 kV/15W maximum power nanofocus X-ray tube and high contrast flat panel detector DXR250 with 2048×2048 pixel, 200×200 µm pixel size.

- Library preparation was performed using a 10x controller (10x Genomics) with the Single Cell 3' v3 chemistry. Sequencing was performed using a HiSeq 3000 (Illumina)

-LC-MS/MS analyses of peptide mixture were done using Ultimate 3000 RSLCnano system connected to Orbitrap Elite hybrid spectrometer (Thermo Fisher Scientific).

- For 3D visualization, the segmentation was done by an operator using a combination of software Avizo 2022.2 (ThermoFisher Scientific) and VG Studio MAX 3.4 (Volume Graphics GmbH, Germany).

Data analysis

Statistical data analysis:
Statistical analysis was done using GraphPad Prism 9 software.

The quantitative data are provided in the Source Data file.

All custom-made scripts used in the analysis are available at: https://github.com/ipoverennaya/RIK_paper

For manuscripts utilizing custom algorithms or software that are central to the research but not yet described in published literature, software must be made available to editors and reviewers. We strongly encourage code deposition in a community repository (e.g. GitHub). See the Nature Portfolio guidelines for submitting code & software for further information.

Data

Policy information about availability of data

All manuscripts must include a data availability statement. This statement should provide the following information, where applicable:

- Accession codes, unique identifiers, or web links for publicly available datasets
- A description of any restrictions on data availability
- For clinical datasets or third party data, please ensure that the statement adheres to our policy

Two knockout mice strains were generated for this manuscript. These will be available upon reasonable request and also be deposited to the Jackson Laboratory. All other relevant data supporting the key findings of this study are available within the article and its Supplementary Information files or from the corresponding author upon reasonable request. The quantitative data generated in this study are provided in the Source Data file. Source data are provided with this paper. The RAW mass spectrometry can be accessed through PRIDE with the identifier PXD039259. RAW Single cell sequencing files can be downloaded from GEO with the accession number GSE206860 [https://www.ncbi.nlm.nih.gov/geo/query/acc.cgi?acc=GSE206860]. RAW Yeat-Two-Hybrid data can be accessed through [https://datadryad.org/stash/share/ojrXiYXvS3yzg5SZwdrXHoggtgeQBDaSgPvh RBdU8Yw].

Human research participants

Policy information about studies involving human research participants and Sex and Gender in Research.

Reporting on sex and gender	n/a
Population characteristics	n/a
Recruitment	n/a
Ethics oversight	n/a

Note that full information on the approval of the study protocol must also be provided in the manuscript.

Field-specific reporting

Please select the one below that is the best fit for your research. If you are not sure, read the appropriate sections before making your selection.

[x] Life sciences   [ ] Behavioural & social sciences   [ ] Ecological, evolutionary & environmental sciences

For a reference copy of the document with all sections, see nature.com/documents/nr-reporting-summary-flat.pdf

Life sciences study design

All studies must disclose on these points even when the disclosure is negative.

Sample size

In our study, we did not perform a formal sample size calculation. Instead, we followed accepted practices in the mouse facilities where the research was conducted to determine the sample size. We initially conducted preliminary experiments to estimate the number of animals needed to achieve adequate statistical power for our tests. Furthermore, the number of animals involved in this research were chosen to reduce unnecessary animal use and to reach statistical significance with two-sided student´s t-test. For the mass spectrometry experiments, we analyzed six independent replicates to ensure the reliability and reproducibility of our findings. For immunofluorescence, single cell sequencing, and transmission electron microscopy, we used three independent samples each. These sample sizes were selected based on the

	same approach of balancing statistical power and accepted practices in the research community to yield robust effects.
Data exclusions	To check whether DE genes between wildtype and knockout are not sex-specific, we compared them with the list of the corresponding DE genes between female and male proximal tubule (PT) samples from (Ransick et al., 2019). The genes whose adjusted p-values are less than 0.01 were excluded from the comparative analysis<br><br>For plotted graphs we did not exclude any data.
Replication	In vitro experiments were repeated a minimum of three times. All attempts were succesful.<br>Parameters related to mice were tested once in several animals (minimum of four). All attempts were succesful.
Randomization	Laboratory animals were allocated to the experimental and control groups based on their genotype and sex.<br>The same accounted for the tissues harvested from these mice.<br>In vitro experiments were allocated based on their treatment (control vs. inhibitor) or transfection condition.
Blinding	Blinding was not applicable to the study because the allocation of laboratory animals to experimental and control groups was based on their genotype and sex, and the tissues harvested from these animals were also allocated accordingly. Additionally, the in vitro experiments were allocated based on treatment (control vs. inhibitor) or transfection condition.

Reporting for specific materials, systems and methods

We require information from authors about some types of materials, experimental systems and methods used in many studies. Here, indicate whether each material, system or method listed is relevant to your study. If you are not sure if a list item applies to your research, read the appropriate section before selecting a response.

Materials & experimental systems

n/a	Involved in the study
☐	☒ Antibodies
☐	☒ Eukaryotic cell lines
☒	☐ Palaeontology and archaeology
☐	☒ Animals and other organisms
☒	☐ Clinical data
☒	☐ Dual use research of concern

Methods

n/a	Involved in the study
☒	☐ ChIP-seq
☒	☐ Flow cytometry
☒	☐ MRI-based neuroimaging

Antibodies

Antibodies used	Mouse monoclonal anti-1700011H14Rik/FAME (B-1) antibody (Santa Cruz, sc-398907, 1:50)<br>Fluorescein-labeled Lotus Tetragonolobus Lectin (Vector Laboratories, FL13212, 1:200)<br>Mouse monoclonal VANGL1 (E-3) antibody (Santa Cruz, sc-166844, 1:200)<br>Chicken polyclonal anti-GFP antibody (Abcam, ab13970, 1:250)<br>Normal Mouse IgG (1µg) (12-371, Merck)<br>Alexa Fluor 555, Donkey anti-Mouse IgG secondary antibody (Invitrogen, A-31570, 1:1000)<br>Alexa Fluor 647, Donkey anti-Chicken IgY secondary antibody (Invitrogen, A-78952, 1:1000)
Validation	1700011H14RIK: Overexpression of GFP-tagged 1700011H14RIK in HEK293 cells and subsequent staining using Alexa 568 Antibody. In addition we stained KO and WT kidneys and found a specific signal in WT memebranal structures in kidney cells versus no specific signal in the KO condition (For Details see Supplementary Figure 21)<br><br>Lotus tetragonolobus lectin: Lotus tetragonolobus lectin (LTL) encompasses a family of closely related glycoproteins with similar specificities toward α-linked L-fucose-containing oligosaccharides. Although many of the binding properties of Lotus lectin are similar to those of Ulex europaeus lectin I (UEL I), the binding affinities and some specificities for oligosaccharides are significantly different between these fucose-specific lectins. This fluorescein-labeled LTL features a ratio of fluorophores to lectin protein that provides optimal staining (excitation 495 nm, emission 515 nm). Supplied as a solution essentially free of unconjugated fluorophores, it is preserved with sodium azide. The recommended inhibiting/eluting sugar is 50-100 mM L-fucose.<br>https://doi.org/10.1016/j.celrep.2022.110473<br><br>Anti-VANGL1: Vangl1 Antibody is a mouse monoclonal IgG1 κ Vangl1 antibody provided at 200 µg/ml specific for an epitope mapping between amino acids 281-298 within a cytoplasmic domain of Vangl1 of human origin. Vangl1 Antibody is recommended for detection of Vangl1 of mouse, rat and human origin by WB, IP, IF and ELISA; also reactive with additional species, including and equine, canine, bovine and porcine<br>https://doi.org/10.1371/journal.pgen.1007840<br><br>anti-GFP: Chicken polyclonal antibody to GFP with over 2500 references: https://www.abcam.com/products/primary-antibodies/gfp-antibody-ab13970.html?productWallTab=ShowAll

	Normal Mouse IgG: Routinely evaluated by IP/WB as a negative non-specific IgG control.<br><br>Alexa Fluor secondary antibodies (Invitrogen): Products have been extensively tested within our own facilities in applications such as cell imaging, flow cytometry and/or fluorescent western blotting. Robust QC procedures guarantee high performance for each individual product. https://www.abcam.com/secondary-antibodies/validated-alexa-fluor-secondaries-for-guaranteed-performance

Eukaryotic cell lines

Policy information about cell lines and Sex and Gender in Research

Cell line source(s)	HEK293T (https://www.atcc.org/products/crl-3216) - newly purchased from ATCC<br>A549 (https://www.atcc.org/products/ccl-185) - validated
Authentication	A549 cells were authenticated by Mycrosynth, Austria. Profiling of the human cell lines was done using highly polymorphic short tandem repeat loci (STRs). STR loci were amplified using the PowerPlex® 16 HS System (Promega). Fragment analysis was done on an ABI3730xl (Life Technologies) and the resulting data were analyzed with GeneMarker HID software (Softgenetics). "The analyzed data of the submitted sample match 100 % to the DNA profile of the cell line A549 (ATCC® CRM-CCL-185™) and 100 % over all 15 autosomal STRs to Microsynth's reference DNA profile of A549 (Mic_150733)."
Mycoplasma contamination	All cell lines were tested negative for mycoplasma
Commonly misidentified lines<br>(See ICLAC register)	HEK293T and A549

Animals and other research organisms

Policy information about studies involving animals; ARRIVE guidelines recommended for reporting animal research, and Sex and Gender in Research

Laboratory animals	FVB/Ant (0 and 75 days) and C57BL/6NCrl (75 days)
Wild animals	No wild animals were used in this study.
Reporting on sex	In our manuscript we used both males and females. These were identified by the animal caretakers. In our manuscript we report some sex specific parameters suggesting that FAME might have a sex-specific role.
Field-collected samples	No field collected samples were used in this study.
Ethics oversight	All animal work was approved and permitted by the Local Ethical Committee on Animal Experiments and conducted according to the Guidelines for Animal Experimentation recommendations (ARRIVE guidelines). In particular, mouse work related to C57Bl/6NCrl was approved and permitted by the Institute of Molecular Genetics of the Czech Academy of Sciences (licence: 45/2017 AVCR). Mouse work related to FVB/Ant by the BMBWF-V/3b (Animal experimentation and genetic engineering, Austria) (licence: BMWFW-66.009/0018-WF/V/3b/2017).

Note that full information on the approval of the study protocol must also be provided in the manuscript.
